# High-Quality Conductive Network Films Constructed from Carbon Nanotube/Carbon Nanofiber Composites via Electrospinning for Electrothermal Applications

**DOI:** 10.3390/nano14201646

**Published:** 2024-10-14

**Authors:** Hedong Huang, Hao Pu, Junwei Fan, Haoxun Yang, Yunhe Zhao, Xinyi Ha, Ruiyun Li, Defeng Jiao, Zeyu Guo

**Affiliations:** College of Materials Science and Art Design, Inner Mongolia Agricultural University, Hohhot 010018, China; huanghedongzml@163.com (H.H.); 15935823029@163.com (J.F.); 15924494917@163.com (H.Y.); zhaoyunh159@163.com (Y.Z.); 18526469994@163.com (X.H.); 15633122786@163.com (R.L.); cyyjxb@163.com (D.J.)

**Keywords:** carbon nanofibers, carbon nanotubes, electrospinning, conductivity, electrothermal properties

## Abstract

In this study, carbon nanotube (CNT)/carbon nanofiber (CNF) composite electrothermal films were prepared by electrospinning, and the effects of the CNT content and carbonization temperature on the electrothermal properties of the CNT/CNF composite films were investigated. The experimental results demonstrated that the conductivity of the CNT/CNF composite electrothermal film (0.006–6.89 S/cm) was directly affected by the CNT content and carbonization temperature. The electrothermal properties of the CNT/CNF positively correlated with the CNT content, carbonization temperature, and applied voltage. The surface temperature of CNT/CNF can be controlled within 30–260 °C, and continuously heated and cooled 100 times without any loss. The convective heat transfer with air is controllable between 0.008 and 31.75. The radiation heat transfer is controllable between 0.29 and 1.92. The prepared CNT/CNF exhibited a heat transfer efficiency of up to 94.5%, and melted a 1 cm thick ice layer within 3 min by thermal convection and radiation alone.

## 1. Introduction

An increasing number of scientists are paying more attention to energy conservation and environmental protection [1]. The traditional method of heating is to burn fossil fuels, such as oil and coal, to obtain heat; however, this process inevitably produces harmful substances (carbon dioxide and solid waste) that damage the environment [2,3]. To improve energy efficiency and reduce environmental pollution, China has implemented a large-scale “coal-to-electricity” policy [4,5]. By independently using electric heating equipment [6,7,8], electricity can be converted into heat energy, thereby effectively reducing environmental pollution and promoting the development of a low-carbon society. Traditional electrothermal materials are mainly metal-based. For example, Cu- and Fe-Cr-Al-based alloy electrothermal materials have a wide range of applications [9,10,11,12]. However, metal-based electrothermal materials have a high cost, low heating efficiency, and complex preparation process, and easily corrode by acid and alkalis [13,14]. In comparison, nonmetal-based electrothermal materials, such as carbon-based electrothermal materials, have become excellent candidates for replacing metal-based electrothermal materials owing to their light weight, excellent thermal and electrical conductivities, and high electrothermal efficiency [15,16,17]. Carbon-based electrothermal materials are typically prepared by dispersing carbon nanoparticles such as graphene, carbon black, carbon nanotubes (CNTs), or other highly conductive nanoparticle fillers into a substrate [18,19]. Graphene and CNTs have recently gained significant attention as the main fillers and are the focus of research regarding carbon materials, with 40.35% of them being graphene and 35.09% being CNTs [20]. However, several chemicals are used in the preparation of graphene oxide, which can cause serious explosion hazards and environmental pollution [21,22]. 

Owing to the unique characteristics of CNTs, including a light weight, high aspect ratio, large specific surface area, high strength and modulus, excellent electrical and thermal conductivity, and impressive chemical stability, they have become the ideal material of electric heating elements [23,24,25,26]. CNTs can be divided into single-walled (SWCNT) and multiwalled (MWCNT) according to the number of layers [27,28]. Owing to the strong intermolecular forces and chemical inertia between the CNTs, they are difficult to disperse and easily form bundles or clusters, which limits their large-scale application in electric heating [29,30]. To solve this problem, it is necessary to functionalize the surface of CNTs to achieve their uniform distribution in different solutions [31,32,33,34]. Carboxylated MWCNTs are stable in different solvents [35]. CNTs have been previously combined with polymer substrates to prepare electrothermal composites. For example, Yang et al. [36] blended CNTs with aramid fibers to achieve a uniform distribution of CNTs in the composite, and the highest surface temperature of the electrothermal material can reach 203 °C; however, this process requires a high input voltage. Xu et al. [37] used a chemical vapor deposition method to prepare CNT films that achieved a maximum surface temperature of 206 °C at an input voltage of 2 V. However, its large-scale application is limited by its complex preparation process and high cost. Therefore, it is necessary to develop new methods to produce large-scale CNT composites with high electrothermal conversion efficiencies.

The electrospinning process [38,39,40,41,42,43] proposed in this study does not strictly depend on the addition of numerous CNTs with highly conductive particles. They are easy to handle, simple, and suitable for large-scale practical applications. A trace amount of CNT doping was found to form an effective conductive path for current transmission in the substrate material. The multi-interface interaction forces formed by the hydrogen bonding and π-π stacking were combined to form the dimensional/nanoscale networks between the CNTs and carbon nanofibers (CNFs) of the substrate material [28,44]. This combination does not require the addition of other chemicals or modification of CNTs, thus significantly saving resources and avoiding environmental pollution. According to the previous research work [45,46], the electrothermal properties of carbon nanofibers were successfully enhanced by adding carbon powder and titanium carbide into the carbon nanofiber matrix, respectively. The introduction of carbon powder helps to increase the number of conductive paths, thus improving the electrical conductivity of the fiber as a whole; the thermal conductivity of carbon nanofibers was also improved. This method provides a powerful support for further optimizing the performance of carbon nanofibers in electrothermal applications, and shows its broad application prospects in the field of intelligent heating materials. A high distribution of hydroxylated MWCNTs in CNFs can be achieved by using a uniform distribution of hydroxylated MWCNTs in organic solvents. CNT/CNF composites exhibit excellent electrical conductivity. The surface temperature can be directly controlled by adjusting the external voltage and amount of CNTs. The unique feature of this study is that a low-cost, environment-friendly, and high-efficiency electrospinning method has been developed, and at the same time, the high-efficiency electrical conductivity and electrothermal properties of micro-carbon nanotube-doped materials have been successfully controlled, providing innovative solutions for future aerospace and high-tech applications.

## 2. Experimental Section

### 2.1. Materials

The main reagents used in this study were as follows. The MWCNTs (hydroxyl functionalized, reagent grade, inner diameter of 3–5 nm, outer diameter of 8–15 nm, 50 um length) were purchased from Shandong Xiya Co., Ltd. (Linyi, China). Polyacrylonitrile (PAN-(C_3_H_3_N)_n_, average molecular weight of 150,000, CSA:25014-41-9, Lot#:C14894372) was purchased from Shanghai Mclean Biochemical Technology Co., Ltd. (Shanghai, China). *N,N*-dimethyl amide (DMF) was purchased from Tianjin Windboat Chemical Reagent Technology Co., Ltd. (Tianjin, China). All of the chemicals were directly used without further purification.

### 2.2. Preparation of CNT/CNF

**Figure 1:**
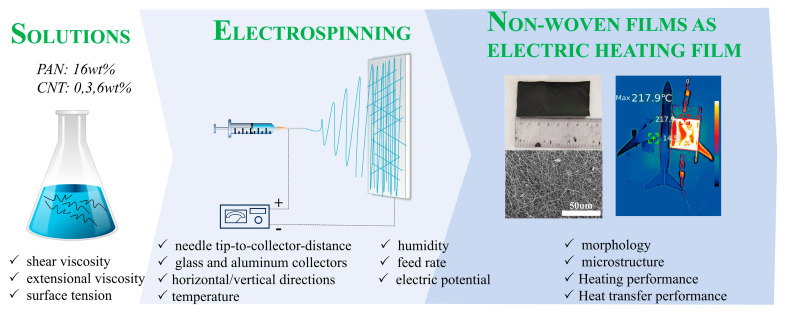
Schematic of the CNT/CNF synthesis.

The CNT/CNF composites were successfully prepared by electrospinning, the process of which is shown in Figure 1. First, different proportions of CNTs (0%, 3%, and 6% by weight relative to PAN) were dissolved in DMF at room temperature and then ultrasonicated for 30 min to obtain a uniform solution. Subsequently, 16% of PAN was added to the above solution, and the mixture was stirred at 60 °C for 24 h to prepare a uniform polymer solution for electrospinning. Table 1 lists the numbers and names of the recipes.

The CNT/PAN composites were prepared by electrospinning dispersed PAN and CNT nanoparticles in DMF. During electrospinning, the needle tip and collecting device used were 18 cm long, the voltage was 15 kV, and the injection speed was 0.5 mm/min.

The CNT/PAN composites obtained by electrospinning were heat-treated at 280 °C in air and subjected to pre-oxidation for 2 h. The specific heating rates and names are listed in Table 2.

In the carbonization stage, the pre-CNT/CNF samples were treated in a nitrogen atmosphere at 800 °C, 900 °C, 1000 °C, and 1100 °C, respectively, obtaining the CNT/CNF composites. The specific heating rates and names are listed in Table 3.

### 2.3. Characterization

Field-emission scanning electron microscopy (SEM; SU8010, Hitachi, Tokyo, Japan) was used to observe the micromorphologies of the CNTs and CNT/CNFs. The chemical structures of CNT/PAN, Pre-CNT/CNF, and CNT/CNF were analyzed using Fourier transform infrared spectroscopy (FTIR, Spectrum 65, Perkin Elmer, Waltham, MA, USA). The chemical structures of CNT/CNF at different carbonization temperatures were analyzed using Raman spectroscopy (Raman, inVia^TM^, Renishaw, Wotton-under-Edge, UK). The conductivity of the CNT/CNF composite (under extreme conditions and at different carbonization temperatures) was analyzed using a four-probe resistance tester (ST-2258C, Suzhou Lattice Electronics Co., Ltd., Suzhou, China). The surface temperature of the CNT/CNF composite was analyzed using a transient temperature meter (TR230X, NAPU Technology, Shanghai, China) with a test time of 1 min to record the readings, and a power supply for the student power (RPS3003D-2). The thermal imaging infrared thermograms of the CNT/CNF were analyzed using an infrared thermal imager (TMi280S, Uni-trend Technology (China) Co., Ltd., Dongguan City, China). The geometric angle coefficients of the CNT/CNF composites were calculated using COMSOL Multiphysics 6.1 (COMSOL, Stockholm, Sweden)**.**

## 3. Results and Discussion

### 3.1. Chemical Structures of CNT/PAN, Pre-CNT/CNF, and CNT/CNF

**Figure 2:**
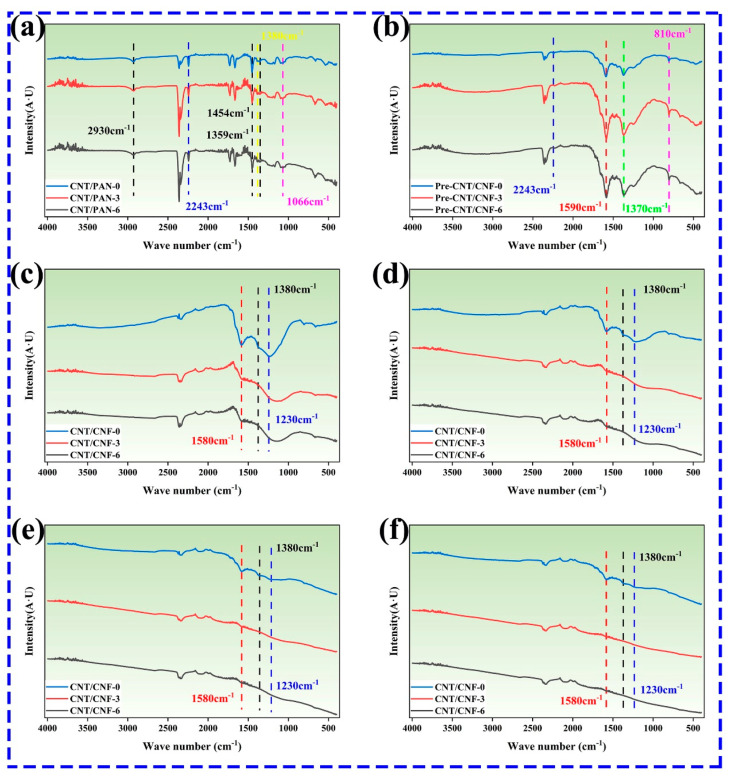
FTIR spectra of (**a**) CNT/PAN, (**b**) pre-CNT/CNF, (**c**) CNT/CNF (800 °C), (**d**) CNT/CNF (900 °C), (**e**) CNT/CNF (1000 °C), and (**f**) CNT/CNF (1100 °C).

**Figure 3:**
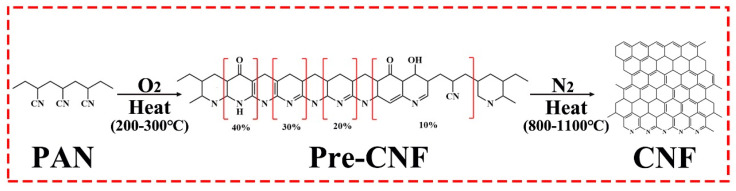
Flow chart of molecular formula changes of PAN, pre- CNF, and CNF during pre-oxidation and carbonization.

The analyzed FTIR results of the chemical structures of CNT/PAN, pre-CNT/CNF, and CNT/CNF are shown in Figure 2. For CNT/CNF, the infrared characteristic peaks mainly include -C≡N, -CH_2_, -CH, and -C-CN [47,48,49,50,51], located at 2243, 1359, 1454, 2930, 1380, and 1066 cm^−1^, respectively, as shown in Figure 2a. The main chemical reactions that occurred in the subsequent pre-oxidation process were cyclization, dehydrogenation, and oxidation [52]. A schematic of the transition process of the chemical molecules is shown in Figure 3. The macroscopical color of CNT/PAN was black (the PAN film was white, which turned black when CNT was added) and gradually changed to brownish yellow; at this time, the internal functional groups, mainly -C≡N, began to break and form -C=N, thus -C=N and -C=C formed a conjugate structure.

The FTIR spectra of the pre-CNT/CNF are shown in Figure 2b. The peak strength of -C=N at 2243 cm^−1^ decreases, and the peaks at 1590 cm^−1^ and 810 cm^−1^, corresponding to -C=N and -C=C, respectively, are consistent with the results above. In the late stages of pre-oxidation, the main chemical reactions are dehydrogenation and oxidation. During dehydrogenation, the brownish-yellow color of the pre-CNT/CNF gradually changed to black. During the oxidation reaction, C-O-C and O=N in the CNT/PAN were oxidized to C=O and -CONH. However, there was no strict order of cyclization, dehydrogenation, or oxidation during the pre-oxidation process. At the end of the pre-oxidation phase, the pre-CNT/CNF samples contained acridine (40%), pyridine (30%), hydrogenated naphthalene (20%), and aromatic heterocyclic ring structures (10%) [53], as shown in Figure 3. During the subsequent carbonization phase, nearly all of the chemical functional groups on the surface of the pre-CNT/CNF were decomposed by heat, whereas -C=C and -C-C were present at 1580 cm^−1^ and 1230 cm^−1^, as well as weak -CH at 1380 cm^−1^, as shown in Figure 3. As the carbonization temperature increased from 800 °C to 1100 °C, all types of hetero-atoms were gradually eliminated, and the aromatic ring condensation rearrangement reaction was initiated. In the infrared spectrum, the intensity of -C=C- and -C-C- further decreased, indicating that the degree of carbonization tended to be complete. Figure 2c, d demonstrate that the characteristic peaks of all of the functional groups nearly completely disappeared during this process. Note that CNT is a carbon material and does not change its chemical structure at high temperatures; therefore, there is no discussion regarding CNTs during heat treatment.

**Figure 4:**
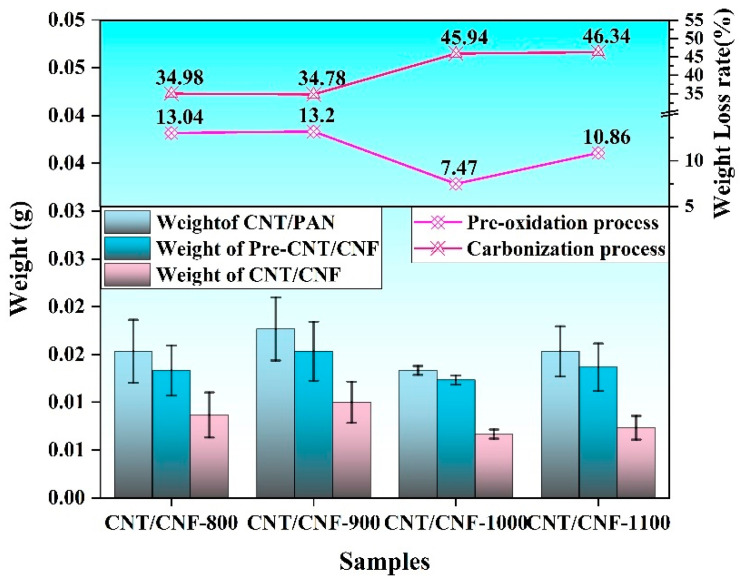
Weight and weight-loss rate during heat treatment.

Figure 4 presents the mass loss during the heat treatment. During the transition from CNT/PAN to pre-CNT/CNF (CNT content: 6%), the main byproducts were H_2_, H_2_O, CH_4_, and NH_3_, which were discharged as gases into the exhaust system. CNTs have excellent high temperature resistance, showing very high thermal stability and heat resistance. Carbon nanotubes can withstand temperatures up to 2800 °C in a vacuum or inert atmosphere, and about 600 °C in air. In addition, carbon nanotubes have a theoretical melting point of 3500 °C and a near-zero coefficient of thermal expansion, which means they have excellent thermal expansion stability with minimal size changes at high temperatures. The quality loss rate was approximately 10%, as shown in Figure 4. PAN experienced dehydrogenation, ammonia, and water release, as well as cyano cyclization to form a primary aromatic structure, resulting in a mass loss of 5–10%. At this stage, ammonia, water, HCN, and other small molecular gases are the main reasons leading to weight loss. In the transformation process of pre-CNT/CNF into CNT/CNF, the main by-products were small-molecule gases such as NH_3_, H_2_O, and HCN; during this process, the rate of quality loss gradually increased (39.84–46.34%), which is consistent with the infrared spectroscopy results.

### 3.2. Microstructure of CNT/CNF

**Figure 5:**
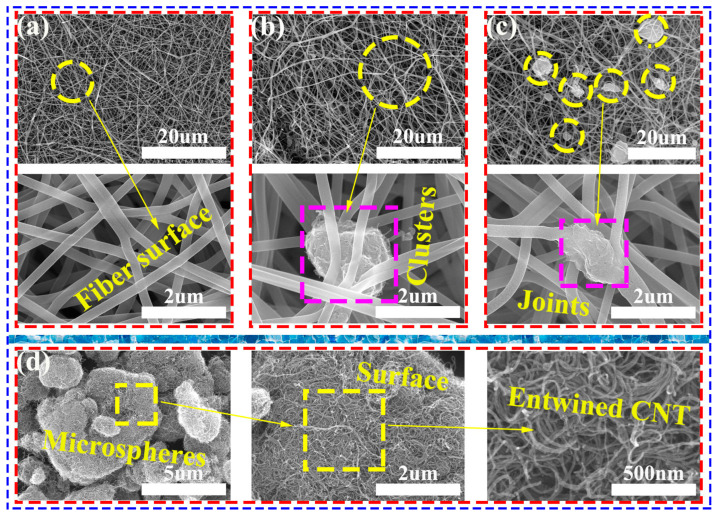
SEM images of (**a**) CNT/CNF-0, (**b**) CNT/CNF-3, (**c**) CNT/CNF-6, and (**d**) CNT.

Figure 5 summarizes the SEM images of the microscopic distribution of CNT in the CNF (the carbonation temperature of the sample is 1100 °C). Each image, in turn, presents low- and high-power SEM images of the CNT/CNF. The microtopography of CNT/CNF-0 is shown in Figure 5a, where the fiber surfaces are smooth and jumbled, forming pores between the fibers. The fibers did not appear to break. When the CNTs were slightly doped, some of the CNTs formed clusters owing to their intermolecular forces or strong Johannes Diderik van der Waals forces, as shown in Figure 5b.

Fiber breakage was also observed among the carbon nanofibers, and a small number of single CNTs were distributed on the surface or inside the carbon nanofibers. Certain CNTs formed “joint-like” structures inside the carbon nanofibers, as shown in Figure 5c. As the CNT content increased, the probability and number of CNT clusters or joint-like structures significantly increased, as shown in Figure 5a–c. The pure CNT powder was also analyzed by SEM, and it was found that a single CNT existed as CNT microspheres in the natural state, and the surfaces of the CNT were interlaced and entwined, as shown in Figure 5d. Owing to the hydrogen and π-π bonds between CNF and CNT, CNT can be easily pasted onto the surface or inside the CNF. Although the distribution of these heterotypic structures within the CNF is random, certain single CNTs will interweave with CNF, which will further promote the conductivity of CNF. The relationship between the CNT/CNF conductivity and CNT content is further discussed in the next section. However, because CNTs are stable in DMF, the mechanical agitation of PAN promotes the formation of aggregates of partial CNTs, which are randomly distributed in the CNF substrate material. These aggregates form a true three-dimensional conductive network within the fiber, which promotes a faster electron flow and thus enhances the conductivity of CNF to a certain extent. Note that the shape and size of the CNT also play a role; for the common conductive nanoparticles, the surface area of the filler is generally considered to have better electrical conductivity. For example, the sheet-like and fibrous conductive nanoparticles have a more complete conductive network than the spherical conductive nanoparticles because they have a larger area to build a conductive network inside the substrate. Generally, we believe that the number of conductive particles has a significant impact on the conductivity of the CNT/CNF; the two are positively correlated. However, this is limited in an actual situation. For example, CNT/CNF is the main substrate material for CNT/CNF, which is also the main support for its mechanical properties. Thus, it cannot be applied in real life. Therefore, a good balance between the performance and utility is needed. Simultaneously, considering the high cost of CNTs, we only selected a small amount of CNT doping in this study.

### 3.3. Conductivity of CNT/CNF

**Figure 6:**
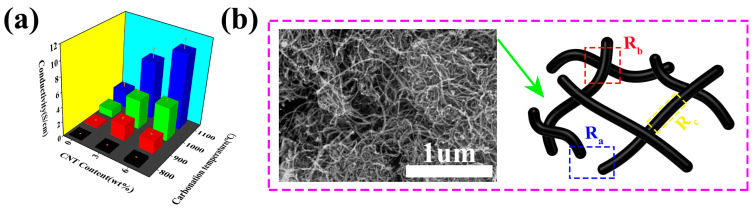
(**a**) Conductivity values of CNFs with different CNT contents and carbonization temperatures. (**b**) Conductive path models of CNT/CNF.

CNTs play an important role in the structure of CNF and constitute a rich conductive network. Nine types of CNT/CNF composites were prepared by adding different amounts of CNT to CNF at different carbonization temperatures, demonstrating different electrical properties, as shown in Figure 6. The conductivity of CNT/CNF-0, CNT/CNF-3, and CNT/CNF-6 were 0.006, 0.18, and 0.39 S/cm, respectively, when the carbonization temperature was 800 °C.

When the carbonization temperature was 900 °C, the conductivities of CNT/CNF-0, CNT/CNF-3, and CNT/CNF-6 were 0.30, 2.15, and 1.78 S/cm, respectively.

When the carbonization temperature was 1000 °C, the conductivities of CNT/CNF-0, CNT/CNF-3, and CNT/CNF-6 were 1.07, 4.06, and 4.62 S/cm, respectively. When the carbonization temperature was 1100 °C, the conductivities of CNT/CNF-0, CNT/CNF-3, and CNT/CNF-6 were 2.31, 7.82, and 10.32 S/cm, respectively. The experimental results demonstrate that the conductivity of CNT/CNF positively correlates with the CNT content and carbonization temperature. Generally, when conductive particles in the substrate material contact one another to form a conductive circuit, a current is formed [54]. When the conductive particles are not in direct contact with one another, the carriers can stimulate the flow of the electric current under the action of a strong electric field [55]. The resistance produced by the uncontacted parts of the CNT is called the tunnel resistance R_a_, that produced by the cross-linked parts is called the contact resistance R_b_, and that of a single CNT is its resistance R_c_. Their values are indicated by M_1_, M_2_, and M_1_ + M_2_ + 1, respectively; the conductive model is shown in Figure 6b. Assuming that there are N such conductive paths in CNT/CNF and that they do not interfere with one another in parallel, the resistance R_i_ of a single conductive path and the total resistance R in CNT/CNF are as follows:

R_i_ = R_a_ M_1_ + R_b_ M_2_ + R_c_(M_1_ + M_2_ + 1) (1)

R = R_i_/N = [R_a_ M_1_ + R_b_ M_2_ + R_c_(M_1_ + M_2_ + 1)]/N (2)

Assuming that the effective area of a single CNT producing the tunnel resistance is S and the voltage between the electrodes is V, the tunnel resistance Ra [56] is as follows:

(3) $$R_{a}=V/\mathrm{JS}=\frac{8\pi h\omega }{3S\Upsilon e^{2}}\exp(\Upsilon \omega )$$

where Υ = $\frac{4\pi }{h}\sqrt{2m\varphi }$, J is the tunneling current, e is the electron charge, m is the electron mass, ω is the thickness of the polymer matrix barrier, φ is the height of the tunneling barrier, and h is Planck’s constant.

To simplify the model, ignoring R_a_ and R_b_, the total resistance between the electrodes is R:

(4) $$R=\frac{R_{a}M_{1}}{N}=\frac{M_{1}8\pi h\omega }{3\mathrm{NS}\Upsilon e^{2}}\exp(\Upsilon \omega )$$

Based on Formula (4), the number of conductive paths N in the CNT/CNF is the only controllable variable. When the amount of CNT doping increases, the number of conductive paths N also increases. A smaller R value results in a greater conductivity. According to this experimental model, the relationship between the CNT content and CNT/CNF conductivity can be qualitatively explained, which is in agreement with the experimental results. The actual physical conduction process is highly complex, and the aforementioned model has certain shortcomings. For example, the conduction paths formed by the CNT are unparallel and do not disturb one another; therefore, there must be different degrees of crosslinking. The conductive model simplifies this process.

**Figure 7:**
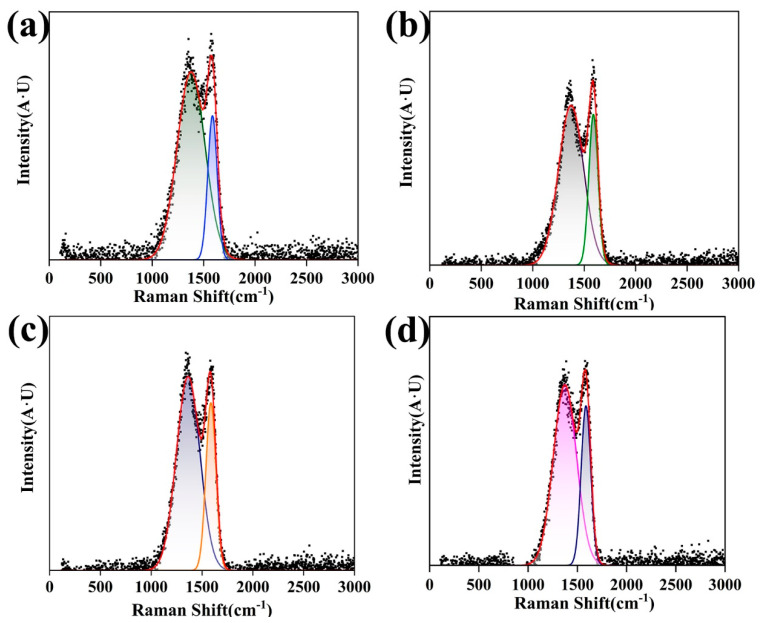
(**a**–**d**) Raman spectra of CNT/CNF-6 prepared at different carbonization temperatures (800–1100 °C).

Figure 7 presents the Raman spectra of CNT/CNF-6 at different carbonization temperatures [57]. In the Raman spectra at 0–3000 cm^−1^, there are broad main bands (D and G peaks) at 1350 and 1380 cm^−1^. The former corresponds to the disordered structure of the sample and represents disordered or defective carbon [58,59], and the latter corresponds to the ordered structure of graphite carbon and represents ordered carbon or that participating in SP^2^ hybridization [60,61]. When the carbonization temperature was 800 °C, the CNT/CNF contained numerous heteroatoms and several defects, which limit the flow of carriers. When the carbonization temperature gradually increased (900–1100 °C), the non-carbon elements were further excluded, the carbon atoms were reorganized, perfecting the SP^2^ structure, the degree of graphitization of the CNT/CNF tended to be complete, and the defects were significantly reduced; at this point, the number of carriers passing through and the probability notably increased. The conductivity of CNT/CNF is typically evaluated by calculating the strength area (I_D_/I_G_) of the D and G bands [62,63]. A smaller I_D_/I_G_ value results in a higher crystallinity of the graphite and better conductivity. Details regarding the Raman spectra of CNT/CNF-6 are presented in Table 4. As the carbonization temperature increased, the I_D_/I_G_ value of CNT/CNF-6 decreased from 3.88 to 2.14. A higher crystallinity of the graphite results in a better electrical conductivity. This result is consistent with the experimental results.

**Figure 8:**
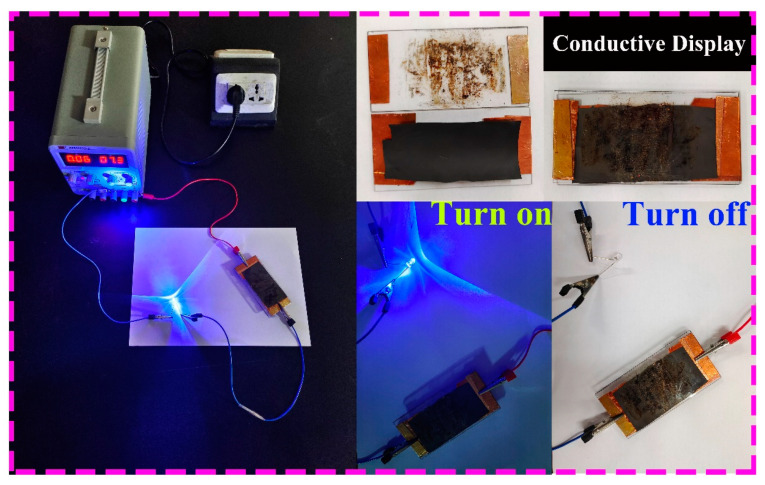
Demonstration of the conductive pathway in the CNT/CNF membrane. The LED light illuminates when the circuit is closed, indicating good electrical conductivity of the CNT/CNF composite membrane.

Simultaneously, to detect the application of CNT/CNF in real-life, CNT/CNF-6 was placed in a closed-loop circuit. When the circuit was disconnected, the LED lamp was not lit; when the circuit was connected, the LED lamp was lit. This proves that CNT/CNF has excellent conductivity and can be used in real-life applications, as shown in Figure 8.

**Figure 9:**
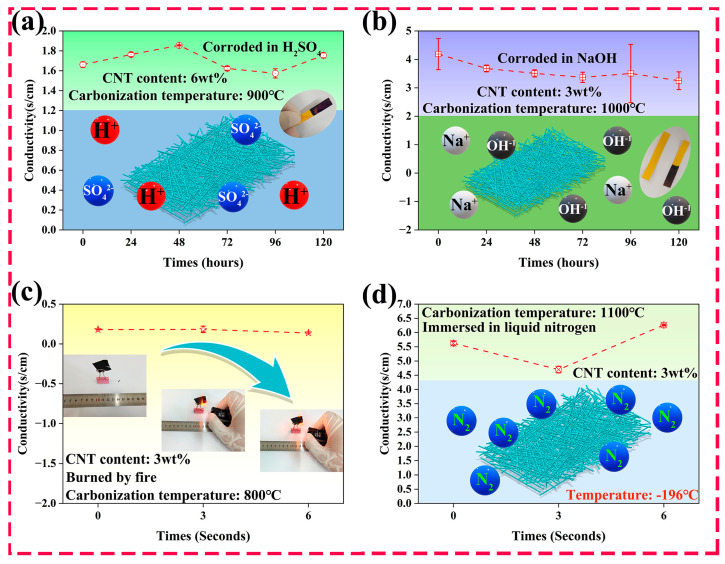
Conductivity of CNT/CNF (**a**) under a strong acid treatment [H_2_SO_4_ (pH = 1)], (**b**) under a strong alkali treatment [NaOH (pH = 14)], (**c**) after high-temperature burning [1300 °C], and (**d**) after cryogenic freezing [−196 °C].

The conductivity of the CNT/CNF composites was tested under extreme conditions. After soaking CNT/CNF-6 (900 °C) in concentrated sulfuric acid for more than 120 h, the electrical conductivity of CNT/CNF-6 remained stable within 1.57–1.82 S/cm without a large numerical fluctuation, which proves its excellent acid corrosion resistance, as shown in Figure 9a. After soaking CNT/CNF-3 (1000 °C) in a strong alkali for more than 120 h, the electrical conductivity of CNT/CNF-3 remained stable within 3.24–4.18 S/cm, which demonstrates that CNT/CNF has an excellent alkali corrosion resistance in an alkaline environment, as shown in Figure 9b.

CNT/CNF-3 (800 °C) underwent a short-term combustion, and a short-term immersion of CNT/CNF-3 (1100 °C) was conducted at low temperatures, with fluctuations of only 0.05 and 1.57 S/cm in the conductivity values, as shown in Figure 9c, d. This proves that CNT/CNF can maintain a stable conductivity at high and low temperatures. The main reason for these phenomena is that the CNT/CNF is mainly composed of carbon materials, which have a strong chemical inertia and low chemical activity, and thus cannot easily react with chemical reagents. Therefore, the CNT/CNF prepared in this study maintained an excellent conductivity in extreme environments (strong acids, strong bases, high temperatures, and low temperatures). This potential can be maintained for future industrial applications.

### 3.4. Electrothermal Properties of CNT/CNF

**Figure 10:**
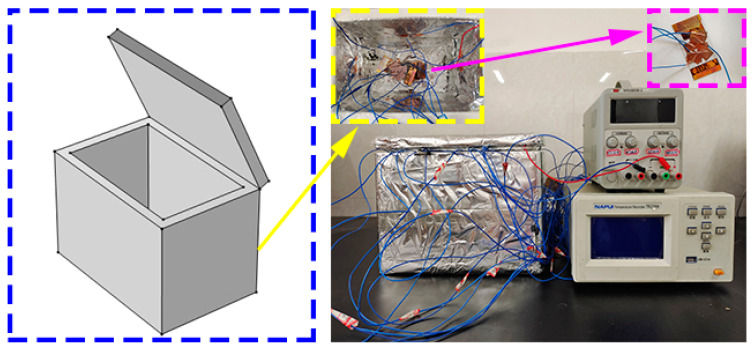
Electro-thermal characteristic test system for the electro-thermal film.

The electrothermal performance of the CNT/CNF composite was tested using a self-made electrothermal performance test system consisting of three parts: a power supply, temperature-measuring box, and surface temperature-measuring instrument, as shown in Figure 10. The output voltages of the power supply were 0, 6, 12, and 18 V. The temperature-measuring box was mainly composed of intumescent polyester (outer: 29.5 cm × 20 cm × 19.5 cm, inner: 25.5 cm × 18 cm × 16 cm). The inner and outer surfaces were covered with a double-layer aluminum foil for heat insulation. Temperature sensors were randomly arranged on the surface of the CNT/CNF composite and inside the temperature-measuring box to monitor the temperature change in real-time.

**Figure 11:**
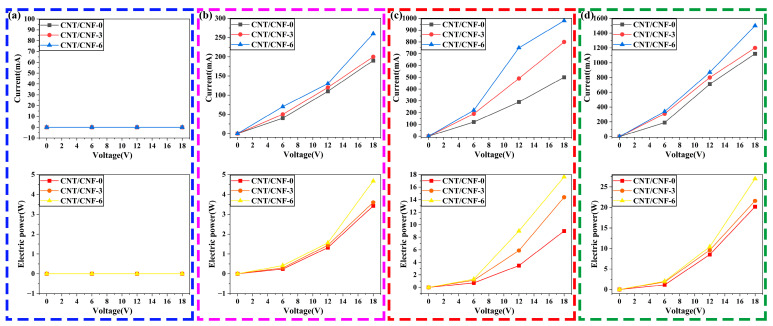
(**a**–**d**) Relationship between the voltage, current, and power of CNT/CNF at different carbonization temperatures (800–1100 °C).

Figure 11 presents the *U-I* and *U-P* curves of the CNT/CNF with different CNT contents and carbonization temperatures when connected to a closed circuit. At the same voltage, the current of the CNT/CNF with the CNT filler was higher than that of the CNT/CNF without the CNT additive alone. According to Ohm’s law, the resistivity of the CNT/CNF with the CNT additive was lower, which is consistent with the aforementioned experimental results in terms of conductivity. The electric powers of the CNT/CNF composites with different CNT contents were calculated from the detected current and applied voltage. Similar to the aforementioned conclusion, the electric power of the CNT/CNF with CNT was clearly higher than that of the CNT/CNF without CNT. At a carbonization temperature of 800 °C, the input voltages were 0, 6, 12, and 18 V, and the current was 0 mA for all of the samples, as shown in Figure 11a. The main reason for this phenomenon is that the carbonization temperature was too low, the resistance value was too large, and the current was unable to smoothly pass through the CNT/CNF; therefore, the closed circuit did not present the current value. In addition, the U-I curves of the other CNT/CNFs demonstrated good linear independence, indicating that the electrical transport properties of these composite films obeyed Ohm’s law. The power and input voltages exhibited a quadratic-exponential relationship. CNT/CNF exhibited Ohmic behavior at low currents, and the linear behavior of CNT/CNF demonstrated a slight deviation at high currents [64].

When a voltage was applied to the CNT/CNF, the input electrical energy was converted to internal (Q_c_) and thermal (φ) energies. When the electric energy of the input CNT/CNF changed to Q_c_, the temperature of the CNT/CNF increased. When the temperature of CNT/CNF was greater than the ambient temperature, the CNT/CNF began to pass heat conduction (φ_h_) to the outside, and the convection (φ_c_) and thermal radiation (φ_t_) were exothermic. The surface temperature of CNT/CNF did not increase when the electric and exothermic energies were in dynamic equilibrium. According to Joule’s law, the quantity of heat generated by the electrified CNT/CNF is Q:

(5) $$\begin{array}{c} Q=\mathrm{Pt}=I^{2}\mathrm{Rt}=\frac{U^{2}}{R}t=Q_{c}+\Phi =\mathrm{mC}\left(t_{2}-t_{1}\right)+\Phi_{h}+\Phi_{c}+\Phi_{t}\\ =\mathrm{mC}\left(t_{2}-t_{1}\right)+\lambda \frac{A}{\delta }\left(t_{2}-t_{1}\right)+\mathrm{hA}\left(t_{2}-t_{3}\right)+C_{1}\left[{\left(\frac{T_{2}}{100}\right)}^{4}-{\left(\frac{T_{4}}{100}\right)}^{4}\right]\mathrm{At} \end{array}$$

Here, P is the power of the CNT/CNF at electrification (W), R is the resistance of CNT/CNF (Ω), U is the voltage of CNT/CNF at electrification (V), t is the electrification time of CNT/CNF (h), m is the mass of CNT/CNF (kg), t_2_ is the real-time temperature of CNT/CNF (°C), t_1_ is the initial temperature of CNT/CNF (°C), C is the heat capacity of CNT/CNF (J/K), λ is the thermal conductivity of the quartz plate (W/m·k), δ is the thickness of the quartz plate (m), h is the convective heat transfer surface coefficient, t_3_ is the real-time air temperature (°C), C_1_ is the system radiation coefficient, T_2_ is the real-time absolute temperature (K) of the quartz plate, and T_4_ is the real-time absolute temperature (K) of the inner wall of the temperature-measuring box.

During the experiment, the surface temperature of the CNT/CNF cannot be directly measured; therefore, it is necessary to directly test the surface of the quartz plate in direct contact with the CNT/CNF, ignoring the heat dissipation of the quartz plate. The surface temperature of the default quartz plate is the same as that of the CNT/CNF, which only conducts heat to the upper surface of the quartz plate. The heat exchange between the CNT/CNF and the outside was ignored.

According to Formula (5), the surface temperature (t_2_) of the CNT/CNF, air temperature inside the temperature-measuring box (t_3_), and size of the inner wall of the temperature-measuring box (T_4_) are only related to the voltage (U), resistance (R), and access time (t) of the CNT/CNF.

#### 3.4.1. Thermal Conductivity of CNT/CNF

**Figure 12:**
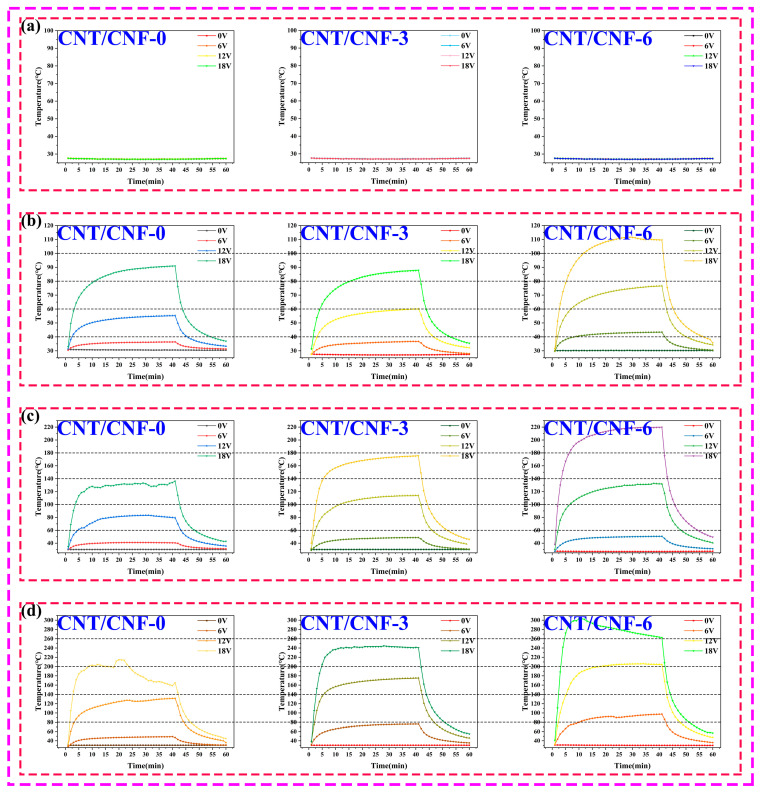
(**a**–**d**) The heating temperature of CNF at different carbonization temperature (800–1100 °C) changes with the electrifying time.

**Figure 13:**
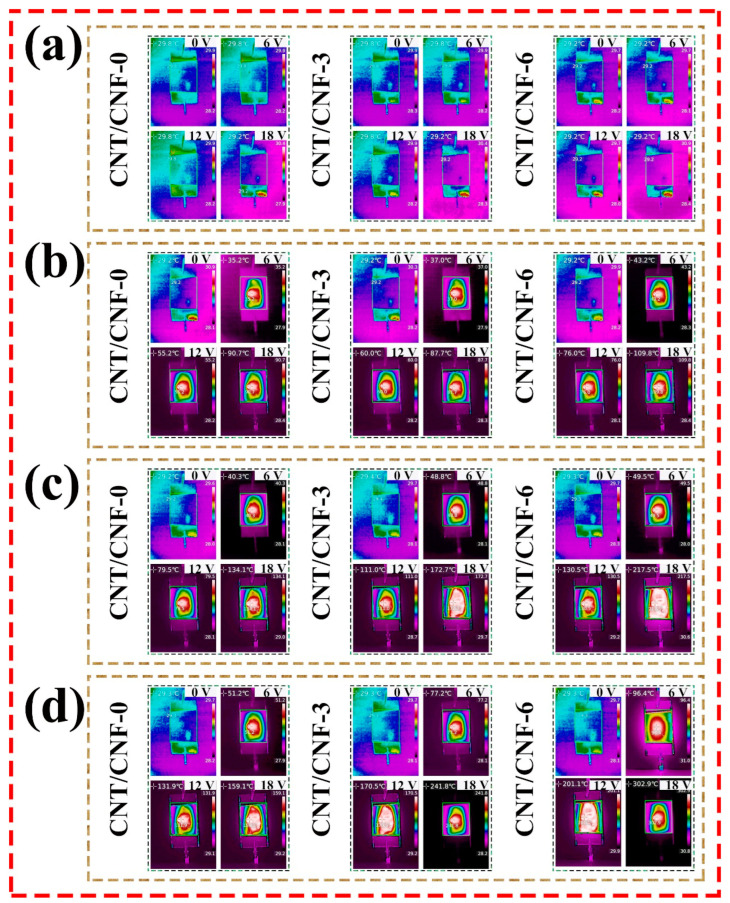
(**a**–**d**) The instantaneous thermal infrared temperature of carbonized CNT/CNF at 800–1100 °C.

The surface temperature of the CNT/CNF composite was measured after low-voltage (0–18 V) connections at room temperature. The experimental results are shown in Figure 12. When the carbonization temperature was 800 °C, the CNT/CNF had numerous non-carbon elements, the conductivity was low, the resistance was large, and the current was unable to smoothly pass through the CNT/CNF, and thus was unable to produce heat with a large joule value, as shown in Figure 12a. When the carbonization temperature was 900 °C and electrification was conducted for 40 min, the maximum surface temperatures of CNT/CNF-0 were 30.5, 36.3, 55.2, and 90.8 °C at the input voltages of 0, 6, 12, and 18 V, respectively. The maximum surface temperatures of CNT/CNF-3 were 27.0, 36.7, 60.2, and 87.7 °C at the input voltages of 0, 6, 12, and 18 V, respectively. The maximum surface temperatures of CNT/CNF-6 were 30.2, 43.3, 76.5, and 109.6 °C at the input voltages of 0, 6, 12, and 18 V, respectively. When the carbonization temperature was 1000 °C and electrification time was 40 min, the maximum surface temperatures of CNT/CNF-0 were 30.2, 40.5, 79.7, and 134.0 °C at the input voltages of 0, 6, 12, and 18 V, respectively. The maximum surface temperatures of CNT/CNF-3 were 30.2, 48.6, 113.9, and 175.2 °C at 0, 6, 12, and 18 V, respectively, and those of CNT/CNF-6 were 30.2, 48.6, 113.9, and 175.2 °C at 0, 6, 12, and 18 V, respectively; the maximum surface temperatures were 29.9, 48.6, 137.8, and 210.6 °C. When the carbonization temperature was 1100 °C and the electrification time was 40 min, the maximum surface temperatures of CNT/CNF-0 were 30.2, 49.1, 131.28, and 159.1 °C at input voltages of 0, 6, 12, and 18 V, respectively. The maximum surface temperatures of CNT/CNF-3 were 30.2, 76.3, 175.2, and 241.3 °C at 0, 6, 12, and 18 V, respectively, and those of CNT/CNF-6 were 30.2, 76.3, 175.2, and 241.3 °C at 0, 6, 12, and 18 V, respectively; the maximum surface temperatures were 30.2, 90.6, 203.5, and 260.5 °C. The experimental results demonstrate that the surface temperature of the CNT/CNF increases as the CNT content and carbonization temperature increase when the applied voltage and electrification time remain the same. According to Formula (5), the surface temperature of the CNT/CNF is only related to the electrification time, applied voltage, and resistance. When the electrification time was the same as that of the applied voltage, regardless of whether the CNT content or carbonization temperature increased, the conductivity of the CNT/CNF decreased its resistance and led to an increase in its surface temperature. When the resistance of the CNT/CNF was the same as the applied voltage, according to Joule’s law, the surface temperature positively correlated with the electrification time within a certain range. The surface temperature of the CNT/CNF increased with the applied voltage when its resistance was consistent with the electrifying time. Figure 13 presents an infrared image of CNT/CNF at the same power-on time, confirming the conclusion above. The infrared image also exhibits a uniform heat dissipation owing to the uniform distribution of CNT in CNF; the infrared image also presents a uniform heat dissipation.

The time-dependent curve of the surface temperature is shown in Figure 12. The curve can be divided into three regions: temperature increase (rapid heating) (0–10 min), quasi-equilibrium (10–40 min), and cooling (40–60 min) [65,66,67]. In the temperature-increase phase, the increase in temperature over time can be empirically expressed as follows [67,68]:

(6) $$\left(\frac{T_{\left(t\right)}-T_{0}}{T_{m}-T_{0}}\right)=1-e^{-t/\tau }$$

where τ is the characteristic growth constant, which is used to express the heating efficiency of the sample, and T_(t)_, T_0_, and T_m_ are the real-time, initial, and highest surface temperatures of the CNT/CNF, respectively. The τ value for the aforementioned CNT/CNF was calculated by fitting data from the first stage (t = 0–10 min) of the curve of the temperature versus time, which is juxtaposed in Table 5.

The results demonstrate that the τ value of CNT/CNF slightly decreased as the applied voltage, CNT content, and carbonization temperature increased. Overall, a low τ value indicates that CNT/CNF has a rapid temperature response to the applied voltage. The mass of CNT/CNF also affects the τ values to a certain extent; generally, more heat is absorbed by a larger mass, thus more time is needed for the heat conversion and temperature increase [69]. In conclusion, CNT/CNFs containing CNT fillers can be successfully used to prepare electrically heated materials owing to their rapid temperature response and effective electrical power at a given applied voltage. Furthermore, the maximum temperature of CNT/CNF can be controlled by adjusting the composition of the CNT.

**Figure 14:**
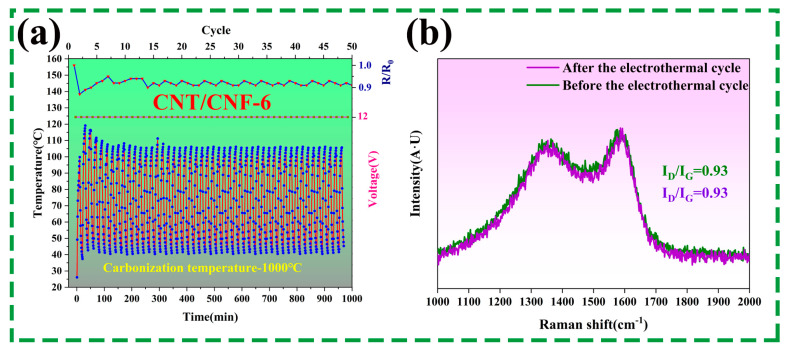
(**a**) Time–temperature curves of CNT/CNF-6 under a 12 V DC voltage applied with repeated heating and cooling cycles and R/R_0_. (**b**) Raman spectra before and after the electrothermal cycle.

Note that stability is the most important factor in electrically heated materials. The thermal stability of the CNT/CNF composite was tested by using the temperature–time curve of the heating/cooling cycle at 12 V. As shown in Figure 14a, the surface temperature sharply increased at a voltage of 12 V, reaching the maximum temperature starting from room temperature in a significantly short time, and then cooled to room temperature when the power supply was disconnected.

Most importantly, the initial/real-time resistances (R/R_0_) did not fluctuate over a wide range during the 100 heating/cooling cycles, proving that CNT/CNF has the ability to continuously undergo cyclic heating significantly stably. Figure 14b depicts the Raman spectra before and after the electrothermal cycle, demonstrating a nearly overlapping Raman spectra and no change in the I_D_/I_G_ values, indicating that the disordered and ordered structures in the CNT/CNF were not affected by the heating cycle.

#### 3.4.2. Convective Analysis of CNT/CNF

Based on the aforementioned data, we assume that only convective heat transfer occurs between CNT/CNF and the air inside the temperature-measuring box, the value of which is Φ_c_ = hA(t_2_ − t_3_) according to Formula (5). During the experiment, the differences among the air temperature, CNT/CNF temperature (t_2_ − t_3_), and CNT/CNF surface area (A) were calculated. Therefore, the default mode of convection between the CNT/CNF and air inside the temperature-measuring box is self-heating in a large space h as follows:

(7) $$h=0.15\lambda [\frac{g(t_{2}-t_{3})}{v^{2}(273+t_{m})}{]}^{1/3}{\mathrm{Pr}}^{1/3}$$

The parameters in the experimental process are introduced in Formula (7) for its derivation. The calculation process is detailed in Supporting Information S1. The calculations of h and Φ_c_ are listed in Table 6.

As shown in Table 6, the h and Φ_c_ values of CNT/CNF positively correlated with the carbonization temperature, applied voltage, and CNT content. This is because as more electrons pass through the CNT/CNF, the heat transfer to air increases, which is also consistent with the results of the thermal conductivity analysis.

#### 3.4.3. Effects of the Thermal Radiation of CNT/CNF

**Figure 15:**
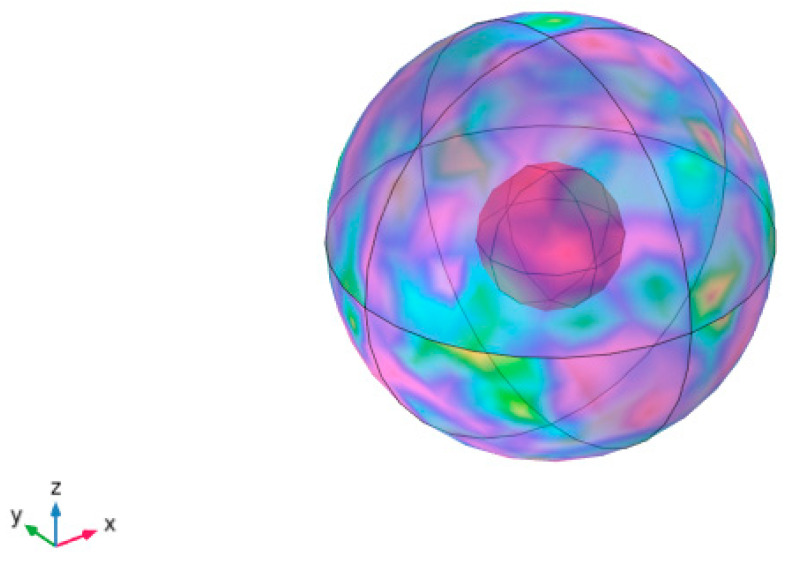
Heat radiation diagram of CNT/CNF.

Regarding the CNT/CNF connection voltage, the CNT/CNF only radiated the inner wall of the thermometer, assuming that the inner walls of the CNT/CNF and thermometer were concentric hollow balls, as shown in Figure 15. The thermal radiation heat of CNT/CNF was calculated using the gray-body radiation method. In this study, the CNT/CNF was a small hollow ball, and the six sides of the inner wall of the temperature-measuring box were regarded as large hollow balls. The model was then simplified as a radiation heat transfer between the surfaces of the closed system. Then, the net radiation heat transfer between CNT/CNF and the inner wall of the temperature-measuring box Φ_i_ is as follows:

(8) $$\Phi_{i}=\frac{A_{i}\varepsilon_{i}(J_{i}-\sigma_{b}T_{i}^{4})}{\varepsilon_{i}-1}$$

The derivation of Equation (8) is detailed in Supporting Information S2. The geometric angular coefficients of the CNT/CNF and inner walls of the temperature-measuring box were F_1-1_ = 0, F_1-2_ = 0.99, F_2-2_ = 0.91, and F_2-1_ = 0.086. The detailed modeling and calculation processes are presented in Supporting Information S3. The Φ_t_ results of the radiation heat transfer are shown in Table 7.

The radiation heat Φ_t_ of CNT/CNF increased as the carbonization temperature, external voltage, and CNT content increased. This conclusion is consistent with the experimental results of the heat conduction and heat transfer analyses. In fact, the CNT/CNF testing in a temperature box is not strictly insulated, and a certain amount of heat passes through the temperature box to the outside. Simultaneously, the heat transfer between the CNT/CNF and air, and that between the CNT/CNF and inner walls of the temperature-measuring box, is not only heat transfer or heat radiation, but also the result of a mixture of various exothermic methods. Here, we simplify the entire system to systematically validate the results.

#### 3.4.4. Practical Application of the CNT/CNF Electrothermal Film

**Figure 16:**
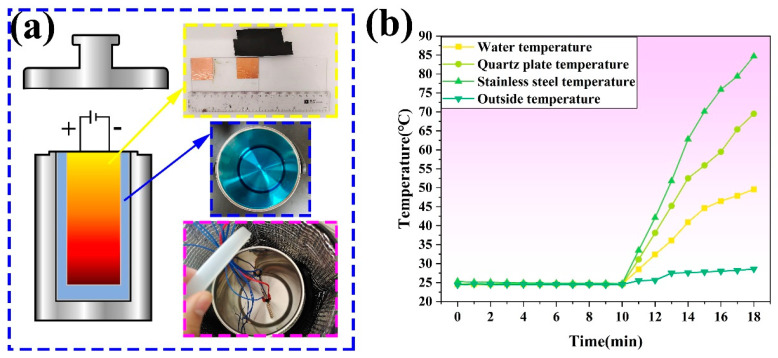
(**a**) Physical diagram of the electrothermal efficiency of the CNT/CNF and the (**b**) electrothermal efficiency.

The CNT/CNF composite was placed in a closed stainless-steel insulated barrel to measure its heat transfer efficiency. The electrothermal efficiency of the CNT/CNF composite was calculated by measuring the temperature difference between the distilled water, stainless-steel inner wall, and quartz plate during electrification, as shown in Figure 16. After 8 min of electrification, the differences in the temperature of the distilled water, stainless steel inner wall, and quartz plate were 25.1, 59.9, and 45 °C, respectively, as shown in Figure 16b. Ignoring the external heat, the default closed stainless steel insulation copper was used for the closed space system. The calculated thermal conversion efficiency of CNT/CNF was 94.5%, which proves that the prepared CNT/CNF has a high electrothermal conversion efficiency and significant application potential in aerospace [70], such as high-altitude icing [71,72] and other fields.

**Figure 17:**
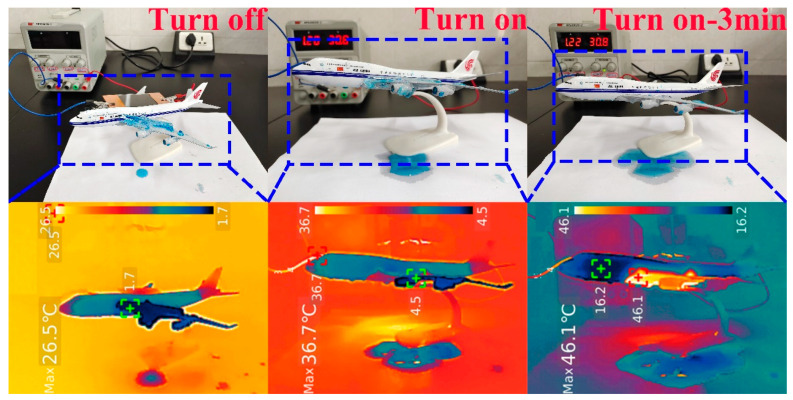
De-icing experiment of CNT/CNF.

To simulate an actual scenario of CNT/CNF icing at high altitudes on an aircraft wing, a 1 cm thick layer of ice was frozen on one wing of an aircraft (frozen water was Methylene blue for easy observation), and CNT/CNF was placed on the other wing; the ice-melting experiments were conducted at high altitudes on aircraft wings, as shown in Figure 17. When the power supply was not connected, the temperature of the aircraft was lower than the ambient temperature. When the power was turned on for 3 min, the ice above the wings of the aircraft completely melted. Note that the CNT/CNF is placed on the non-icing side, as with CNT/CNF on the icing side, the icing time will be further shortened. The CNT/CNF prepared by us has proven to be significantly advantageous for future practical applications.

## 4. Conclusions

Electrospinning technology was used to skillfully insert CNT into the inner surface of CNF. Owing to the excellent physical and chemical properties of CNF, their conductivity and electrothermal properties can be further improved. CNT/CNF does not require a large driving voltage but only a safe voltage (<36 V). It has a significantly safe operating space and an incomparable electrothermal conversion efficiency. The CNF content and carbonization temperature directly affected the conductivity and electrothermal properties of the CNT/CNF composite. The CNT content and carbonization temperature can be adjusted to satisfy the requirements. CNT/CNF can drive a low-voltage LED lamp to light-up in a closed-loop circuit and can de-ice in a significantly short time.

In summary, the prepared CNT/CNF has excellent properties and wide application prospects in electrical conduction and electrothermal applications.

## Figures and Tables

**Table 1:** The composition and name of the sample.

PAN (g)	CNT (g)	DMF (g)	CNT/PAN	PAN/(PAN + DMF)	Name
1.28	0	6.72	0 wt%	16 wt%	CNT/PAN-0
1.28	0.0384	6.72	3 wt%	16 wt%	CNT/PAN-3
1.28	0.0768	6.72	6 wt%	16 wt%	CNT/PAN-6

**Table 2:** Pre-oxidation temperature program and name.

Temperature Stage (°C)	Status	Time (min)	Name	Pre-Oxidation Name
0–250	Duration	60	CNT/PAN-0	Pre-CNT/CNF-0
250–250	Keep warm	60	CNT/PAN-3	Pre-CNT/CNF-3
250–260	Duration	10	CNT/PAN-6	Pre-CNT/CNF-6
260–260	Keep warm	60	--	--
260–270	Duration	10	--	--
270–270	Keep warm	60	--	--
270–280	Duration	10	--	--
280–280	Keep warm	120	--	--

**Table 3:** Carbonization temperatures and names.

Temperature Stage (°C)	Status	Time (min)	Name	Carbonization Name
0–800	Duration	160	Pre-CNT/CNF-0	CNT/CNF-0
800–800	Keep warm	10	Pre-CNT/CNF-3	CNT/CNF-3
0–900	Duration	180	Pre-CNT/CNF-6	CNT/CNF-6
900–900	Keep warm	10	--	--
0–1000	Duration	200	--	--
1000–1000	Keep warm	10	--	--
0–1100	Duration	220	--	--
1100–1100	Keep warm	10	--	--

**Table 4:** Fitting parameters of the Raman spectra for CNT/CNF-6 at different carbonization temperatures.

Carbonization Temperature (°C)	D Band		G Band		(I_D_/I_G_) Two Peak Area Ration
	Peak Position (cm^−1^)	Full Width at Half Maximum (cm^−1^)	Peak Position (cm^−1^)	Full Width at Half Maximum (cm^−1^)	
800	1378.14	319.61	1585.85	107.58	3.88
900	1372.64	287.50	1589.86	105.21	2.89
1000	1363.23	273.55	1587.16	116.36	2.72
1100	1374.14	270.18	1587.32	114.53	2.14

**Table 5:** τ values of CNT/CNF under different conditions.

Carbonization Temperature	Samples	τ			
		0 V	6 V	12 V	18 V
800 °C	CNT/CNF-0	-	-	-	-
	CNT/CNF-3	-	-	-	-
	CNT/CNF-6	-	-	-	-
900 °C	CNT/CNF-0	-	7.91	7.61	7.18
	CNT/CNF-3	-	7.32	7.05	6.95
	CNT/CNF-6	-	6.24	6.35	5.59
1000 °C	CNT/CNF-0	-	5.55	5.52	5.44
	CNT/CNF-3	-	4.98	4.68	6.50
	CNT/CNF-6	-	3.87	3.78	3.11
1100 °C	CNT/CNF-0	-	3.26	3.19	-
	CNT/CNF-3	-	2.15	1.99	1.78
	CNT/CNF-6	-	1.58	1.3	-

**Table 6:** h and Φ_c_ values of CNT/CNF under different conditions.

Carbonization Temperature	Samples	h (W/(m^2^/K))				Φ_c_ (W)			
		0 V	6 V	12 V	18 V	0 V	6 V	12 V	18 V
800 °C	CNT/CNF-0	-	-	-	-	-	-	-	-
	CNT/CNF-3	-	-	-	-	-	-	-	-
	CNT/CNF-6	-	-	-	-	-	-	-	-
900 °C	CNT/CNF-0	-	0.34	0.78	1.94	-	0.008	0.082	0.493
	CNT/CNF-3	-	0.40	1.17	2.58	-	0.017	0.16	0.645
	CNT/CNF-6	-	0.55	1.79	4.75	-	0.026	0.353	1.631
1000 °C	CNT/CNF-0	-	0.59	1.81	5.15	-	0.026	0.364	2.206
	CNT/CNF-3	-	0.75	1.89	6.79	-	0.071	0.440	3.305
	CNT/CNF-6	-	0.77	2.75	-	-	0.076	14.40	-
1100 °C	CNT/CNF-0	-	0.77	4.22	-	-	0.076	2.087	-
	CNT/CNF-3	-	1.82	5.16	18.56	-	0.369	2.202	14.40
	CNT/CNF-6	-	1.96	9.98	34.46	-	0.510	7.835	31.75

**Table 7:** Φ_t_ of CNT/CNF under different conditions.

Carbonization Temperature	Samples	Φt (W)			
		0 V	6 V	12 V	18 V
800 °C	CNT/CNF-0	-	-	-	-
	CNT/CNF-3	-	-	-	-
	CNT/CNF-6	-	-	-	-
900 °C	CNT/CNF-0	-	0.29	0.36	0.51
	CNT/CNF-3	-	0.29	0.38	0.50
	CNT/CNF-6	-	0.31	0.45	0.62
1000 °C	CNT/CNF-0	-	0.30	0.40	0.60
	CNT/CNF-3	-	0.34	0.74	1.34
	CNT/CNF-6	-	0.35	0.67	1.40
1100 °C	CNT/CNF-0	-	0.35	0.77	-
	CNT/CNF-3	-	0.45	0.90	1.47
	CNT/CNF-6	-	0.51	1.52	1.92

## Data Availability

Data and code for the analysis are available upon request from the corresponding author.

## Supplementary Materials

The supporting information can be downloaded at: https://www.mdpi.com/article/10.3390/nano14201646/s1.
