# Supplementary material for: High-Quality Conductive Network Films Constructed from Carbon Nanotube/Carbon Nanofiber Composites via Electrospinning for Electrothermal Applications
Source: Nanomaterials (Basel). 2024 Oct 14;14(20):1646. doi: 10.3390/nano14201646 (PMC11510678; doi:10.3390/nano14201646)
Supplement: Supplementary file 1 [file nanomaterials-14-01646-s001.zip › Supportimg Information/supporting information-1.pdf]

$$Nu=f(Gr \cdot Pr)=C(Gr \cdot Pr)^n \quad (1)$$

$$Nu=\frac{hl}{\lambda} \quad (2)$$

$$Gr=\frac{g\alpha(t_2-t_3)l^3}{v^2} \quad (3)$$

$$t_m=\frac{t_2+t_3}{2} \quad (4)$$

$$\alpha=\frac{1}{273+t_m} \quad (5)$$

In formula (1)-(5), Nu is the Nusselt number; Gr is the Grashof number number; Pr is the Planck number; h is the convective heat transfer surface coefficient ($W/(m^2 \cdot K)$); λ is the thermal conductivity of heated air ($W/(m \cdot K)$); l is the size (m); α is the volume expansion coefficient (1/K); g is gravitational acceleration; t_2-t_3 is the temperature difference between sample and heated air (℃); v is the moving viscosity (m^2/s); t_2 is the film temperature (℃); t_3 is the air temperature (℃); t_m for qualitative temperature (℃).

So according to formula (1)-(5), the convective heat transfer surface coefficient is:

$$h=0.15\lambda[\frac{g(t_2-t_3)}{v^2(273+t_m)}]^{1/3}Pr^{1/3}$$

The convective heat transfer of CNT/CNF is assumed to be heat transfer with the hot surface facing up and the flow pattern is turbulent. The experimental range of $Gr \cdot Pr$ is: $8\times10^6 \sim 8\times10^{11}$, So C=0.15, n=1/3, l=1.6.

Thermophysical properties of dry saturated steam.

t_m (°C)	$\lambda \times 10^2$ (W/(m • °C))	$v \times 10^6$ (m^2/s)	Pr
0	1.83	1655.01	0.815
10	1.88	896.54	0.831
20	1.94	509.90	0.847
30	2.00	303.53	0.863
40	2.06	188.04	0.883
50	2.12	120.72	0.896
60	2.19	80.07	0.913
70	2.25	54.57	0.930
80	2.33	38.25	0.947
90	2.40	27.44	0.966
100	2.48	20.12	0.984
110	2.56	15.03	1.00
120	2.65	11.41	1.02
130	2.76	8.80	1.04
140	2.85	6.89	1.06
150	2.97	5.45	1.08
160	3.08	4.37	1.11
170	3.21	3.54	1.13
180	3.36	2.90	1.15
190	3.51	2.39	1.18
200	3.68	1.99	1.21
210	3.87	1.67	1.24
220	4.07	1.41	1.26
230	4.30	1.19	1.29
240	4.54	1.02	1.33
250	4.84	0.873	1.36
260	5.18	0.752	1.40
270	5.55	0.651	1.44
280	6.00	0.565	1.49
290	6.55	0.492	1.54
300	7.22	0.430	1.61
310	8.06	0.380	1.70
320	8.65	0.336	1.94
330	9.61	0.300	2.24
340	10.70	0.266	2.84
350	11.90	0.234	3.83
360	13.70	0.203	5.34
370	16.60	0.169	15.7
374.15	23.79	0.143	∞
