# Supplementary material for: High-Quality Conductive Network Films Constructed from Carbon Nanotube/Carbon Nanofiber Composites via Electrospinning for Electrothermal Applications
Source: Nanomaterials (Basel). 2024 Oct 14;14(20):1646. doi: 10.3390/nano14201646 (PMC11510678; doi:10.3390/nano14201646)
Supplement: Supplementary file 1 [file nanomaterials-14-01646-s001.zip › Supportimg Information/supporting information-2.pdf]

The six sides of the inner wall of the temperature measuring box are regarded as a large plane, and it is assumed that the large plane is a diffuse gray surface and the air inside the temperature measuring box does not participate in the heat radiation. Then the model is simplified as the radiation heat transfer between the surfaces of the closed system. The CNT/CNF is marked as plane 1, and the inner wall of the temperature measuring box is marked as plane 2.

Therefore, the effective radiation J_i can be expressed as:

$$J_i=\varepsilon_i E_{bi}+(1-\varepsilon_i)\sum_{j=1}^{n} F_{i\text{-}j} J_j,\ \ j=1,\ 2\ldots n \tag{1}$$

In the formula (1), ε_i is the emissivity of the gray system of surface I; E_{bi} is the self-radiation of surface i (W); $F_{i\text{-}j}$ is the geometric angle coefficient of surface i to surface j; J_j is the effective radiation of surface j; and $E_{bi}=\sigma_b T^4$, $\sigma_b(5.67\times10^{-8}W/(m^2\cdot K^4))$ is the blackbody radiation coefficient. The inner wall surface of the temperature measuring box participating in heat radiation heat transfer is marked as plane 1; TiC/CNF is labeled as plane 1; Then n = 2, so formula (2) is:

$$\sum_{j=1}^{2} F_{i\text{-}j} J_j-\frac{J_i}{1-\varepsilon_i}=\frac{\varepsilon_i}{\varepsilon_i-1}\sigma_b T^4 \tag{2}$$

Then formula (2) is written in the form of a matrix:

$$\begin{bmatrix} F_{1\text{-}1}-\frac{1}{1-\varepsilon_1} & F_{1\text{-}2} \\ F_{2-1} & F_{2\text{-}2}-\frac{1}{1-\varepsilon_2} \end{bmatrix}\times\begin{bmatrix} J_1 \\ J_2 \end{bmatrix}=\begin{bmatrix} \frac{\varepsilon_1\sigma_b T_1^4}{\varepsilon_1-1} \\ \frac{\varepsilon_2\sigma_b T_2^4}{\varepsilon_2-1} \end{bmatrix}$$

By solving the equation, we can get the effective radiation quantity J_i of each surface, and the net radiation heat transfer of each surface Φ_i is:

$$\Phi_i=\frac{A_i\varepsilon_i(J_i-\sigma_b T_i^4)}{\varepsilon_i-1} \tag{3}$$
