# Supplementary material for: High-Quality Conductive Network Films Constructed from Carbon Nanotube/Carbon Nanofiber Composites via Electrospinning for Electrothermal Applications
Source: Nanomaterials (Basel). 2024 Oct 14;14(20):1646. doi: 10.3390/nano14201646 (PMC11510678; doi:10.3390/nano14201646)
Supplement: Supplementary file 1 [file nanomaterials-14-01646-s001.zip › Supportimg Information/supporting information-3.pdf]

An example of geometric angle coefficient calculation

Report date	Jan 3, 2024, 7:48:04 PM

Contents

1. Global Definitions **3**

1.1. Parameters 3

2. Component **1** **4**

2.1. Definitions 4

2.2. Geometry 1 7

2.3. Materials 9

2.4. Surface-to-surface radiation .... **Error! Bookmark not defined.**

2.5. Grid 1 32

3. Study**1** **34**

3.1. Steady state .... **Error! Bookmark not defined.**

3.2. Solver Configurations 34

4. Results .... Error! Bookmark not defined.

4.1. Data set .... **Error! Bookmark not defined.**

4.2. Derived value .... **Error! Bookmark not defined.**

4.3. Form .... **Error! Bookmark not defined.**

1 Global Definitions

Date	Sep 13, 2023, 12:23:18 PM

GLOBAL SETTINGS

Name	An example of geometric angle coefficient calculation.mph
Path	C:\Users\Administrator\Desktop\ An example of geometric angle coefficient calculation.mph
Version	COMSOL Multiphysics 6.1 (Build: 252)
Unit system	SI

USED PRODUCTS

Heat Transfer Module
COMSOL Multiphysics
CAD Import Module

COMPUTER INFORMATION

CPU	Intel(R) Core(TM) i5-5200U CPU @ 2.20GHz, 2 cores, 15.9 GB RAM
Operating system	Windows 10

1.1 PARAMETERS

PARAMETER 1

Name	Expression	Value	Description
r_int	0.3[m]	0.3 m	Radius of the inner sphere
r_ext	1[m]	1 m	Outer sphere radius

2 Component 1

Date	Sep 13, 2023, 12:08:23 PM

SETTINGS

Description	Value
Unit system	Same as global system (SI)
Geometry shape function	Automatic

SPATIAL FRAME COORDINATES

First	Second	Third
x	y	z

MATERIAL FRAME COORDINATES

First	Second	Third
X	Y	Z

GEOMETRY FRAME COORDINATES

First	Second	Third
Xg	Yg	Zg

MESH FRAME COORDINATES

First	Second	Third
Xm	Ym	Zm

2.1 DEFINITIONS

2.1.1 Variables

Indicator, Inner Sphere

SELECTION

Geometric entity level	Boundary
Name	Inner Sphere
Selection	Named geom1_csel1_bnd: Geometry geom1: Dimension 2: Boundaries 5–8, 11–12, 14–15

Name	Expression	Unit	Description
ext	0		External surface indicator
int	1		Inner Surface Indicator

Indicator, outer sphere

SELECTION

Geometric entity level	Boundary
Name	Outer sphere
Selection	Named geom1_csel2_bnd: Geometry geom1: Dimension 2: Boundaries 1–4, 9–10, 13, 16

Name	Expression	Unit	Description
ext	1		External surface indicator
int	0		Inner Surface Indicator

2.1.2 Nonlocal Couplings

Integral, Inner Sphere

Coupling type	Integration
Operator name	intop_int

SELECTION

Geometric entity level	Boundary
Name	Inner Sphere
Selection	Named geom1_csel1_bnd: Geometry geom1: Dimension 2: Boundaries 5–8, 11–12, 14–15

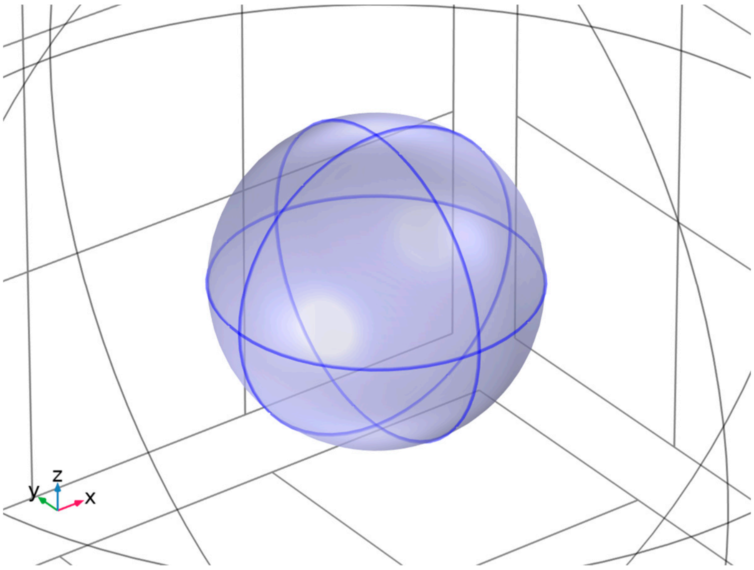

Selection

Integral, outer sphere

Coupling type	Integration
Operator name	intop_ext

SELECTION

Geometric entity level	Boundary
Name	Outer sphere
Selection	Named geom1_csel2_bnd: Geometry geom1: Dimension 2: Boundaries 1–4, 9–10, 13, 16

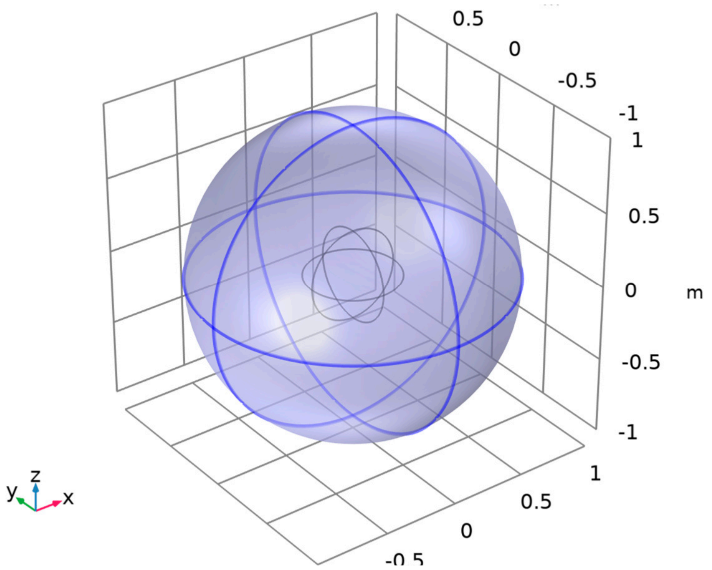

Selection

2.1.3 Coordinate Systems

Boundary coordinate system 1

Coordinate system type	Boundary system
Tag	sys1

COORDINATE NAMES

First	Second	Third
t1	t2	n

2.2 GEOMETRY 1

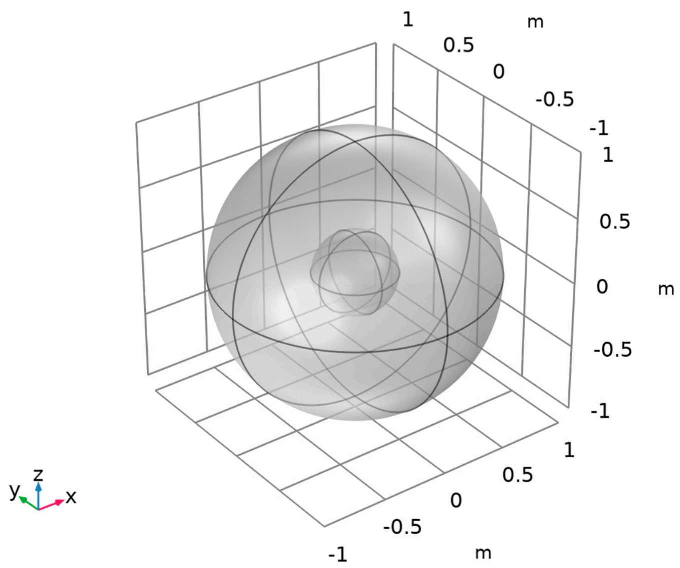

Geometry 1

UNITS

Length unit	m
Angular unit	deg

GEOMETRY STATISTICS

**Description**	**Value**
Space dimension	3
Number of domains	0
Number of boundaries	16
Number of edges	24
Number of vertices	12

2.2.1 sphere 1 (sph1)

SELECTIONS OF RESULTING ENTITIES

**Description**	**Value**
Contribute to	Inner Sphere

POSITION

**Description**	**Value**
Position	{0, 0, 0}

OBJECT TYPE

Description	Value
Type	Surface

AXIS

Description	Value
Axis type	z - axis

SIZE

Description	Value
Radius	r_int

2.2.2 spheres 2 (sph2)

SELECTIONS OF RESULTING ENTITIES

Description	Value
Contribute to	Outer sphere

POSITION

Description	Value
Position	{0, 0, 0}

OBJECT TYPE

Description	Value
Type	Surface

AXIS

Description	Value
Axis type	z - axis

SIZE

Description	Value
Radius	r_ext

2.3 MATERIALS

2.3.1 materials 1

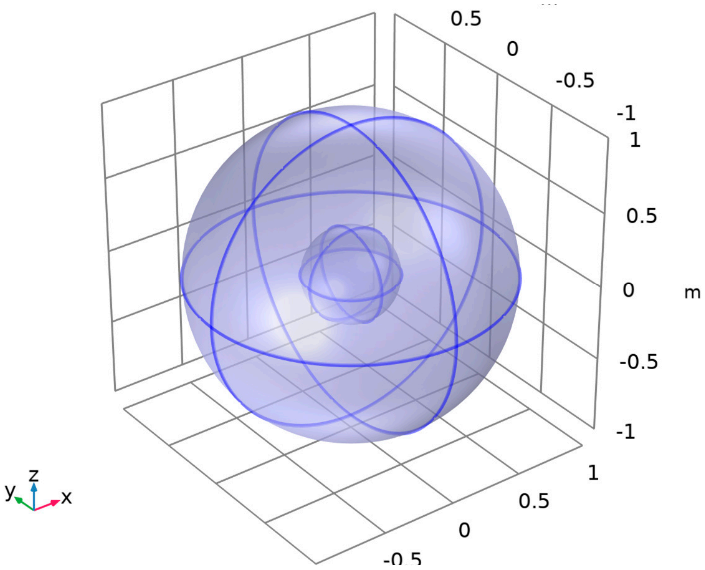

materials 1

SELECTION

Geometric entity level	Boundary
Selection	Geometry geom1: Dimension 2: All boundaries

MATERIAL PARAMETERS

**Name**	**Value**	**Unit**	**Property group**
Surface emissivity	1	1	Basic

BASIC

**Description**	**Value**
Surface emissivity	1

2.4 SURFACE-TO-SURFACE RADIATION

USED PRODUCTS

Heat Transfer Module
COMSOL Multiphysics

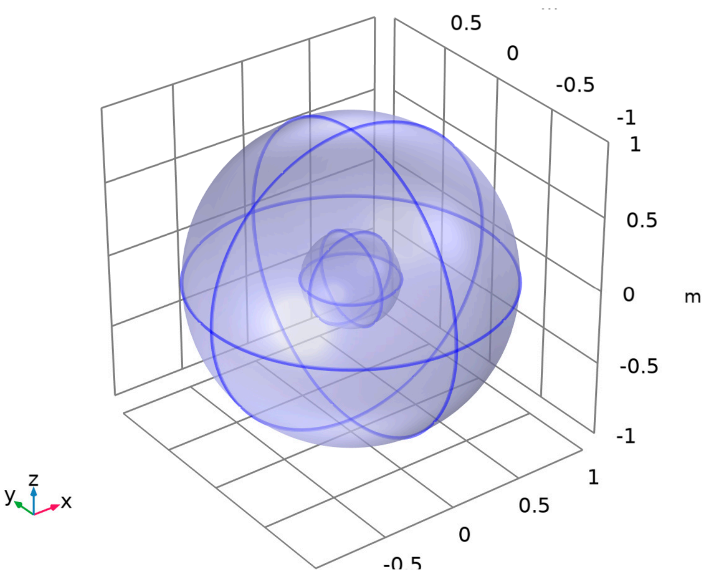

surface-to-surface radiation

SELECTION

Geometric entity level	Boundary
Selection	Geometry geom1: Dimension 2: All boundaries

EQUATIONS

$$J = \varepsilon e_b(T) + \rho_d G$$
$$G = G_m + G_{amb} + G_{ext}$$
$$G_{amb} = F_{amb}\varepsilon_{amb}e_b(T_{amb})$$
$$e_b(T) = n^2\sigma T^4$$

2.4.1 Interface Settings

Discretization

SETTINGS

Description	Value
Surface radiosity	Linear

SETTINGS

Description	Value
Equation form	Study controlled

Radiation Settings

SETTINGS

Description	Value
Jacobian contribution	Only local contributions to radiosity

Description	Value
Use radiation groups	Off
Surface-to-surface radiation method	Hemicube
Radiation resolution	256
Transparent media refractive index	1
Wavelength dependence of radiative properties	Constant
Check consistency	On
Store view factors on disk	Off
View factors update threshold	Every nonlinear iteration

2.4.2 Variables

Name	Expression	Unit	Description	Selection	Details
rad.nx	dnx	1	Normal vector, x-component	Boundaries 1–16	
rad.ny	dny	1	Normal vector, y-component	Boundaries 1–16	
rad.nz	dnz	1	Normal vector, z-component	Boundaries 1–16	
rad.dnx	dnx	1	Normal vector down direction, x-component	Boundaries 1–16	
rad.dny	dny	1	Normal vector down direction, y-component	Boundaries 1–16	
rad.dnz	dnz	1	Normal vector down direction, z-component	Boundaries 1–16	
rad.unx	unx	1	Normal vector up direction, x-component	Boundaries 1–16	
rad.uny	uny	1	Normal vector up direction, y-component	Boundaries 1–16	
rad.unz	unz	1	Normal vector up direction, z-component	Boundaries 1–16	
rad.nS2S	1	1	Transparent media refractive index	Global	
rad.q0su	0	W/m²	Source heat flux	Global	+ operation
rad.q0sd	0	W/m²	Source heat flux	Global	+ operation

Name	Expression	Unit	Description	Selection	Details
rad.dfltopaque	-1	1	Opaque	Boundaries 1–16	
rad.dfltopaque	-1	1	Opaque	Domains -2–0	
rad.opaque	rad.dfltopaque	1	Opaque	Boundaries 1–16	
rad.opaque	rad.dfltopaque	1	Opaque	Domains -2–0	
rad.lambdainf	1[mm]	m	Upper bound for integrations	Boundaries 1–16	
rad.Ts	5780[K]	K	Sun blackbody temperature	Boundaries 1–16	

2.4.3 diffuse reflective surface 1

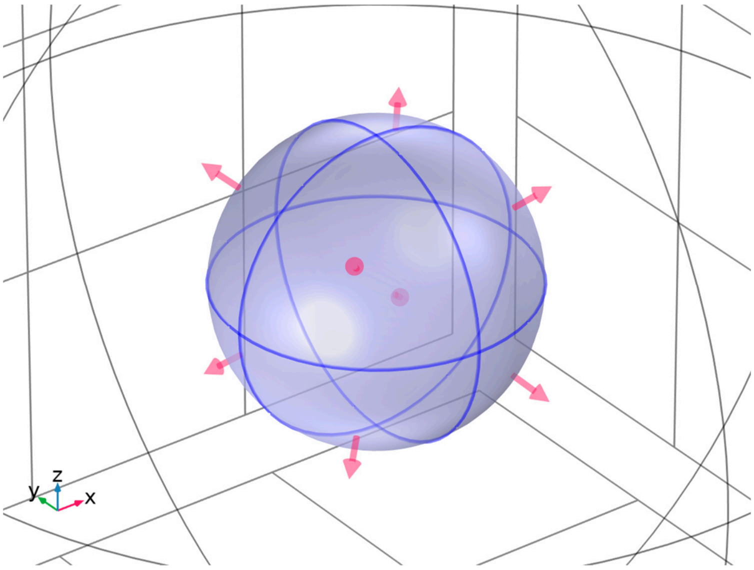

diffuse reflective surface 1

SELECTION

Geometric entity level	Boundary
Selection	Geometry geom1: Dimension 2: All boundaries

EQUATIONS

$$J = \varepsilon e_b(T) + \rho_d G$$

$$\varepsilon + \rho_d = 1$$

$$G = G_m + G_{amb} + G_{ext}$$

$$G_{amb} = F_{amb} e_b(T_{amb})$$

$$G_{ext} = q_s$$

$$e_b(T) = n^2 \sigma T^4$$
$$q_{r,net} = \varepsilon(G - e_b(T))$$

Radiation Direction

SETTINGS

Description	Value
Emitted radiation direction	Positive normal direction

Ambient

SETTINGS

Description	Value	Unit
Define ambient temperature on each side	Off	
Ambient temperature	User defined	
Ambient temperature	293.15	K
Define ambient emissivity on each side	Off	
Ambient emissivity	Blackbody	
Include diffuse irradiance	Off	

Surface Emissivity

SETTINGS

Description	Value
Define properties on each side	Off
Emissivity	From material

Constraint Settings

SETTINGS

Description	Value
Use weak constraints	Off
Constraint method	Elemental

Model Input

SETTINGS

Description	Value	Unit
Temperature	User defined	
Temperature	293.15	K

PROPERTIES FROM MATERIAL

Property	Material	Property group
Surface emissivity	Materials 1	BASIC

Variables

Name	Expression	Unit	Description	Selection	Details
rad.Tradu	rad.Trad	K	Upside temperature	Boundaries 5–8, 11–12, 14–15	
rad.Tradd	rad.Trad	K	Downside temperature	Boundaries 5–8, 11–12, 14–15	
rad.Tamb	293.15[K]	K	Ambient temperature	Boundaries 5–8, 11–12, 14–15	
rad.Famb	rad.Fambu	1	Ambient view factor	Boundaries 5–8, 11–12, 14–15	
rad.Gamb	rad.Gambu	W/m²	Ambient irradiation	Boundaries 5–8, 11–12, 14–15	
rad.Gext	rad.Gextu	W/m²	External irradiation	Boundaries 5–8, 11–12, 14–15	
rad.Gm	rad.Gmu	W/m²	Mutual surface irradiation	Boundaries 5–8, 11–12, 14–15	
rad.Grad_band	rad.Gradu	W/m²	Surface irradiation	Boundaries 5–8, 11–12, 14–15	
rad.Grad	rad.Grad_band	W/m²	Surface irradiation	Boundaries 5–8, 11–12, 14–15	
rad.J	rad.J_band	W/m²	Surface radiosity	Boundaries 5–8, 11–12, 14–15	
rad.J_band	rad.Ju_band	W/m²	Surface radiosity	Boundaries 5–8, 11–12, 14–15	
rad.lambda	1	m	Wavelength	Boundaries 5–8, 11–12, 14–15	
rad.q0s	rad.q0su	W/m²	Source heat flux	Boundaries 5–8, 11–12, 14–15	
rad.rflux	rad.rflux_band	W/m²	Radiative heat flux	Boundaries 5–8, 11–12, 14–15	

Name	Expression	Unit	Description	Selection	Details
rad.rflux_band	rad.rfluxu_band+rad.rfluxd_band	W/m²	Radiative heat flux	Boundaries 5–8, 11–12, 14–15	
rad.zeta	1	1	Intermediate variable	Boundaries 5–8, 11–12, 14–15	
rad.fep	rad.fepu	1	Fractional emissive power	Boundaries 5–8, 11–12, 14–15	
rad.fepamb	rad.fepambu	1	Fractional emissive power	Boundaries 5–8, 11–12, 14–15	
rad.fepdiff	rad.fepdiffu	1	Fractional emissive power	Boundaries 5–8, 11–12, 14–15	
rad.T_band	rad.Tu_band	K	Temperature	Boundaries 5–8, 11–12, 14–15	
rad.T	rad.T_band	K	Temperature	Boundaries 5–8, 11–12, 14–15	
rad.Trad	rad.dsurf1.minput_temperature	K	Temperature	Boundaries 5–8, 11–12, 14–15	
rad.Gm_gp	gpeval(2,rad.Gm)	W/m²	Mutual surface irradiation	Boundaries 5–8, 11–12, 14–15	
rad.Gmu_gp	gpeval(2,rad.Gmu)	W/m²	Mutual surface irradiation, upside	Boundaries 5–8, 11–12, 14–15	
rad.Gmd_gp	0	W/m²	Mutual surface irradiation, downside	Boundaries 5–8, 11–12, 14–15	
rad.Famb_gp	gpeval(2,rad.Famb)	1	Ambient view factor, Gauss point evaluation	Boundaries 5–8, 11–12, 14–15	
rad.Fambu_gp	gpeval(2,rad.Fambu)	1	Ambient view factor, Gauss point evaluation, upside	Boundaries 5–8, 11–12, 14–15	
rad.Fambd_gp	0	1	Ambient view factor, Gauss	Boundaries 5–8, 11–12,	

Name	Expression	Unit	Description	Selection	Details
			point evaluation, downside	14–15	
rad.diru	1	1	Radiation direction, upside	Boundaries 5–8, 11–12, 14–15	
rad.ebu	rad.nS2S^2*sigma_const*rad.Tu_band^4*rad.fepu	W/m²	Blackbody emissive power, upside	Boundaries 5–8, 11–12, 14–15	
rad.ebambu	rad.nS2S^2*sigma_const*rad.Tambu^4*rad.fepambu	W/m²	Blackbody emissive power, upside	Boundaries 5–8, 11–12, 14–15	
rad.fepu	1+15*integrate(rad.zeta^3/(1-exp(rad.zeta)),rad.zeta,eps,eps,1.0E-6)/pi^4	1	Fractional emissive power, upside	Boundaries 5–8, 11–12, 14–15	
rad.fepambu	1+15*integrate(rad.zeta^3/(1-exp(rad.zeta)),rad.zeta,eps,eps,1.0E-6)/pi^4	1	Fractional emissive power, upside	Boundaries 5–8, 11–12, 14–15	
rad.fepdiffu	1+15*integrate(rad.zeta^3/(1-exp(rad.zeta)),rad.zeta,eps,eps,1.0E-6)/pi^4	1	Fractional emissive power, upside	Boundaries 5–8, 11–12, 14–15	
rad.Tu_band	0.5*((1+rad.opaque)*rad.Tradu+(1-rad.opaque)*rad.Tradd)	K	Upside temperature	Boundaries 5–8, 11–12, 14–15	
rad.Gambu	rad.Fambu*rad.epsilon_ambu_band*rad.ebambu	W/m²	Ambient irradiation, upside	Boundaries 5–8, 11–12, 14–15	
rad.Gextu	rad.q0su	W/m²	External irradiation, upside	Boundaries 5–8, 11–12, 14–15	+ operation
rad.Gradu	nojac(rad.Gmu+rad.Gambu+rad.Gextu)	W/m²	Surface irradiation, upside	Boundaries 5–8, 11–12, 14–15	
rad.Ju	rad.Ju_band	W/m²	Surface radiosity, upside	Boundaries 5–8, 11–12, 14–15	

Name	Expression	Unit	Description	Selection	Details
rad.Ju_band	rad.dsurf1.Ju_band	W/m²	Surface radiosity, upside	Boundaries 5–8, 11–12, 14–15	
rad.rfluxu	rad.rfluxu_band	W/m²	Radiative heat flux, upside	Boundaries 5–8, 11–12, 14–15	
rad.rfluxu_band	rad.epsilonu_band*(rad.Gradu-rad.ebu)	W/m²	Radiative heat flux, upside	Boundaries 5–8, 11–12, 14–15	
rad.Idiff	0	W/m²	Diffuse irradiance	Boundaries 5–8, 11–12, 14–15	
rad.Idiff_band	rad.Idiffu_band	W/m²	Diffuse irradiance	Boundaries 5–8, 11–12, 14–15	
rad.Idiffu_band	0	W/m²	Diffuse irradiance, upside	Boundaries 5–8, 11–12, 14–15	
rad.Idiffd_band	0	W/m²	Diffuse irradiance, downside	Boundaries 5–8, 11–12, 14–15	
rad.Tambu	rad.Tamb	K	Ambient temperature, upside	Boundaries 5–8, 11–12, 14–15	
rad.dird	0	1	Radiation direction, downside	Boundaries 5–8, 11–12, 14–15	
rad.ebd	rad.nS2S^2*sigma_const*rad.Td_band^4*rad.fepd	W/m²	Blackbody emissive power, downside	Boundaries 5–8, 11–12, 14–15	
rad.ebambd	rad.nS2S^2*sigma_const*rad.Tambd^4*rad.fepambd	W/m²	Blackbody emissive power, downside	Boundaries 5–8, 11–12, 14–15	
rad.fepd	1+15*integrate(rad.zeta^3/(1-exp(rad.zeta)),rad.zeta,eps,eps,1.0E-6)/pi^4	1	Fractional emissive power, downside	Boundaries 5–8, 11–12, 14–15	
rad.fepambd	1+15*integrate(rad.zeta^3/(1-exp(rad.zeta)),rad.zeta,eps,eps,1.0E-6)/pi^4	1	Fractional emissive power, downside	Boundaries 5–8, 11–12, 14–15	

Name	Expression	Unit	Description	Selection	Details
rad.fepdiffd	1+15*integrate(rad.zeta^3/(1-exp(rad.zeta)),rad.zeta,eps,eps,1.0E-6)/pi^4	1	Fractional emissive power, downside	Boundaries 5–8, 11–12, 14–15	
rad.Td_band	0.5*((1+rad.opaque)*rad.Tradd+(1-rad.opaque)*rad.Tradu)	K	Downside temperature	Boundaries 5–8, 11–12, 14–15	
rad.Gambd	rad.Fambd*rad.epsilon_ambd_band*rad.ebambd	W/m²	Ambient irradiation, downside	Boundaries 5–8, 11–12, 14–15	
rad.Gextd	rad.q0sd	W/m²	External irradiation, downside	Boundaries 5–8, 11–12, 14–15	+ operation
rad.Gradd	nojac(rad.Gmd+rad.Gambd+rad.Gextd)	W/m²	Surface irradiation, downside	Boundaries 5–8, 11–12, 14–15	
rad.Jd	rad.Jd_band	W/m²	Surface radiosity, downside	Boundaries 5–8, 11–12, 14–15	
rad.Jd_band	rad.dsurf1.Jd_band	W/m²	Surface radiosity, downside	Boundaries 5–8, 11–12, 14–15	
rad.rfluxd	rad.rfluxd_band	W/m²	Radiative heat flux, downside	Boundaries 5–8, 11–12, 14–15	
rad.rfluxd_band	0	W/m²	Radiative heat flux, downside	Boundaries 5–8, 11–12, 14–15	
rad.Tambd	rad.Tamb	K	Ambient temperature, downside	Boundaries 5–8, 11–12, 14–15	
rad.epsilon	rad.epsilon_band	1	Emissivity	Boundaries 5–8, 11–12, 14–15	
rad.epsilonu	subst(material.epsilon_rad,rad.dsurf1.minput_temperature,rad.Tradu,rad.dsurf1.minput_length,rad.lambda)	1	Emissivity, upside	Boundaries 5–8, 11–12, 14–15	Meta
rad.epsilond	subst(material.epsil	1	Emissivity,	Boundaries	Meta

Name	Expression	Unit	Description	Selection	Details
	on_rad,rad.dsurf1.minput_temperature,rad.Tradd,rad.dsurf1.minput_length,rad.lambda)		downside	5–8, 11–12, 14–15	
rad.epsilon_band	0.5*((1+rad.opaque)*rad.epsilonu_band+(1-rad.opaque)*rad.epsilond_band)	1	Emissivity	Boundaries 5–8, 11–12, 14–15	
rad.epsilonu_band	integrate(rad.epsilonu,rad.lambda,0[m]*(1+0.25*eps),rad.lambdainf*(1-0.25*eps),1.0E-6)/max(rad.lambdainf*(1-0.25*eps)-0[m]*(1+0.25*eps),eps)	1	Emissivity, upside	Boundaries 5–8, 11–12, 14–15	
rad.epsilond_band	integrate(rad.epsilond,rad.lambda,0[m]*(1+0.25*eps),rad.lambdainf*(1-0.25*eps),1.0E-6)/max(rad.lambdainf*(1-0.25*eps)-0[m]*(1+0.25*eps),eps)	1	Emissivity, downside	Boundaries 5–8, 11–12, 14–15	
rad.epsilon_amb	rad.epsilon_amb_band	1	Ambient emissivity	Boundaries 5–8, 11–12, 14–15	
rad.epsilon_ambu	1	1	Ambient emissivity, upside	Boundaries 5–8, 11–12, 14–15	
rad.epsilon_ambd	1	1	Ambient emissivity, downside	Boundaries 5–8, 11–12, 14–15	
rad.epsilon_amb_band	0.5*((1+rad.opaque)*rad.epsilon_ambu_band+(1-rad.opaque)*rad.epsilon_ambd_band)	1	Ambient emissivity	Boundaries 5–8, 11–12, 14–15	
rad.epsilon_ambu_band	integrate(rad.epsilon_ambu,rad.lambda,0[m]*(1+0.25*e	1	Ambient emissivity, upside	Boundaries 5–8, 11–12, 14–15	

Name	Expression	Unit	Description	Selection	Details
	ps),rad.lambdainf*(1-0.25*eps),1.0E-6)/max(rad.lambdainf*(1-0.25*eps)-0[m]*(1+0.25*eps),eps)				
rad.epsilon_ambd_band	integrate(rad.epsilon_ambd,rad.lambda,0[m]*(1+0.25*eps),rad.lambdainf*(1-0.25*eps),1.0E-6)/max(rad.lambdainf*(1-0.25*eps)-0[m]*(1+0.25*eps),eps)	1	Ambient emissivity, downside	Boundaries 5–8, 11–12, 14–15	

Shape functions

Name	Shape function	Unit	Description	Shape frame	Selection	Details
rad.dsurf1.Ju_band	Lagrange (Linear)	W/m²	Surface radiosity, upside	Spatial	Boundaries 5–8, 11–12, 14–15	
rad.dsurf1.Ju_band	Lagrange (Linear)	W/m²	Surface radiosity, upside	Spatial	Edges 5–8, 12–14, 17–20, 23	Slit
rad.dsurf1.Jd_band	Lagrange (Linear)	W/m²	Surface radiosity, downside	Spatial	Boundaries 5–8, 11–12, 14–15	
rad.dsurf1.Jd_band	Lagrange (Linear)	W/m²	Surface radiosity, downside	Spatial	Edges 5–8, 12–14, 17–20, 23	Slit

Weak Expressions

Weak expression	Integration order	Integration frame	Selection
((1-rad.epsilonu_band)*rad.Gradu+rad.epsilonu_band*rad.ebu-rad.Ju_band)*test(rad.Ju_band)	2	Spatial	Boundaries 5–8, 11–12, 14–15

Constraints

Constraint	Constraint force	Shape function	Selection	Details

Constraint	Constraint force	Shape function	Selection	Details
-rad.Jd_band+rad.Ju_band	-test(rad.dsurf1.Jd_band)	Lagrange (Linear)	Boundaries 5–8, 11–12, 14–15	Elemental

2.4.4 initial value 1

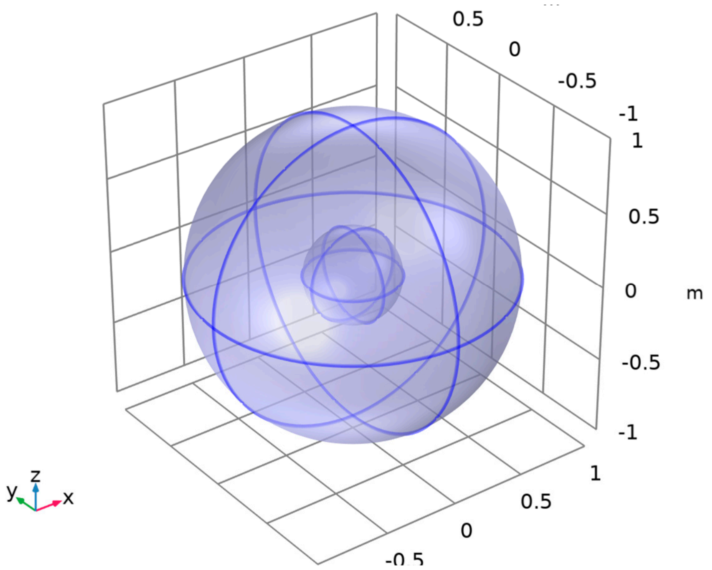

initial value 1

SELECTION

Geometric entity level	Boundary
Selection	Geometry geom1: Dimension 2: All boundaries

EQUATIONS

$$J_{\text{init}} = \varepsilon e_b(T_{\text{init}}) + (1 - \varepsilon)e_b(T_{\text{amb}})$$

Initial Values

SETTINGS

Description	Value	Unit
Initial value	Blackbody/Graybody	
Initial temperature	User defined	
Initial temperature	293.15	K

Variables

Name	Expression	Unit	Description	Selection	Details

Name	Expression	Unit	Description	Selection	Details
rad.init1.Tinit	model.input.Tinit	K	Initial temperature	Boundaries 1–16	Meta
rad.Juinit	subst(rad.epsilonu_band,rad.Tradu,rad.init1.Tinit,rad.Tradd,rad.init1.Tinit)*rad.nS2S^2*sigma_const*rad.init1.Tinit^4+(1-subst(rad.epsilonu_band,rad.Tradu,rad.Tambu,rad.Tradd,rad.Tambd))*rad.ebambu*rad.fepambu	W/m²	Initial surface radiosity	Boundaries 1–16	
rad.Jdinit	subst(rad.epsilond_band,rad.Tradd,rad.init1.Tinit,rad.Tradu,rad.init1.Tinit)*rad.nS2S^2*sigma_const*rad.init1.Tinit^4+(1-subst(rad.epsilond_band,rad.Tradd,rad.Tambd,rad.Tradu,rad.Tambu))*rad.ebambd*rad.fepambd	W/m²	Initial surface radiosity	Boundaries 1–16	
rad.init1.Tinit0	293.15	K	Default initial temperature	Boundaries 1–16	
rad.Tinit	rad.init1.Tinit0	K	Initial temperature	Boundaries 1–16	

2.4.5 diffuse reflective surface 2

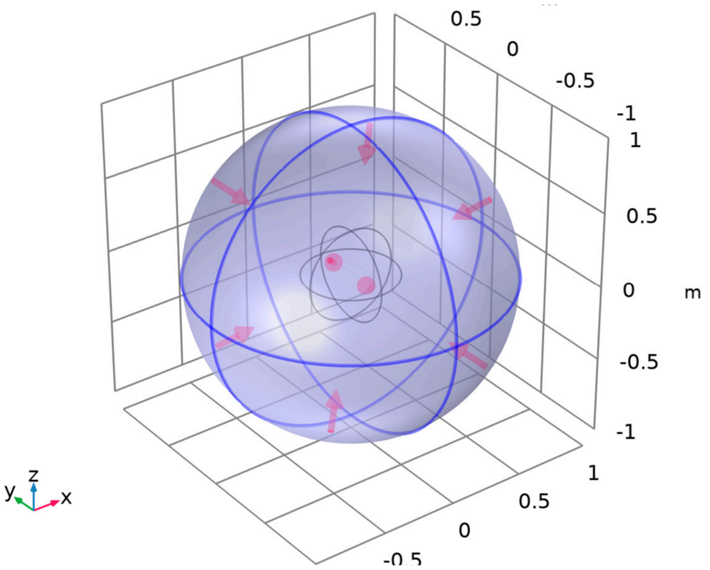

diffuse reflective surface 2

SELECTION

Geometric entity level	Boundary
Name	Outer sphere
Selection	Named geom1_csel2_bnd: Geometry geom1: Dimension 2: Boundaries 1–4, 9–10, 13, 16

EQUATIONS

$$J = \varepsilon e_b(T) + \rho_d G$$
$$\varepsilon + \rho_d = 1$$
$$G = G_m + G_{amb} + G_{ext}$$
$$G_{amb} = F_{amb} e_b(T_{amb})$$
$$G_{ext} = q_s$$
$$e_b(T) = n^2 \sigma T^4$$
$$q_{r,net} = \varepsilon(G - e_b(T))$$

Radiation Direction

SETTINGS

Description	Value
Emitted radiation direction	Negative normal direction

Ambient

SETTINGS

Description	Value	Unit

Description	Value	Unit
Define ambient temperature on each side	Off	
Ambient temperature	User defined	
Ambient temperature	293.15	K
Define ambient emissivity on each side	Off	
Ambient emissivity	Blackbody	
Include diffuse irradiance	Off	

Surface Emissivity

SETTINGS

Description	Value
Define properties on each side	Off
Emissivity	From material

Constraint Settings

SETTINGS

Description	Value
Use weak constraints	Off
Constraint method	Elemental

Model Input

SETTINGS

Description	Value	Unit
Temperature	User defined	
Temperature	293.15	K

PROPERTIES FROM MATERIAL

Property	Material	Property group
Surface emissivity	Materials 1	BASIC

Variables

Name	Expression	Unit	Description	Selection	Details
rad.Tradu	rad.Trad	K	Upside temperature	Boundaries 1–4, 9–10, 13, 16	
rad.Tradd	rad.Trad	K	Downside temperature	Boundaries 1–4, 9–10, 13, 16	
rad.Tamb	293.15[K]	K	Ambient temperature	Boundaries 1–4, 9–10,	

Name	Expression	Unit	Description	Selection	Details
				13, 16	
rad.Famb	rad.Fambd	1	Ambient view factor	Boundaries 1–4, 9–10, 13, 16	
rad.Gamb	rad.Gambd	W/m²	Ambient irradiation	Boundaries 1–4, 9–10, 13, 16	
rad.Gext	rad.Gextd	W/m²	External irradiation	Boundaries 1–4, 9–10, 13, 16	
rad.Gm	rad.Gmd	W/m²	Mutual surface irradiation	Boundaries 1–4, 9–10, 13, 16	
rad.Grad_band	rad.Gradd	W/m²	Surface irradiation	Boundaries 1–4, 9–10, 13, 16	
rad.Grad	rad.Grad_band	W/m²	Surface irradiation	Boundaries 1–4, 9–10, 13, 16	
rad.J	rad.J_band	W/m²	Surface radiosity	Boundaries 1–4, 9–10, 13, 16	
rad.J_band	rad.Jd_band	W/m²	Surface radiosity	Boundaries 1–4, 9–10, 13, 16	
rad.lambda	1	m	Wavelength	Boundaries 1–4, 9–10, 13, 16	
rad.q0s	rad.q0sd	W/m²	Source heat flux	Boundaries 1–4, 9–10, 13, 16	
rad.rflux	rad.rflux_band	W/m²	Radiative heat flux	Boundaries 1–4, 9–10, 13, 16	
rad.rflux_band	rad.rfluxu_band+rad.rfluxd_band	W/m²	Radiative heat flux	Boundaries 1–4, 9–10, 13, 16	
rad.zeta	1	1	Intermediate variable	Boundaries 1–4, 9–10, 13, 16	
rad.fep	rad.fepd	1	Fractional emissive power	Boundaries 1–4, 9–10, 13, 16	

Name	Expression	Unit	Description	Selection	Details
rad.fepamb	rad.fepambd	1	Fractional emissive power	Boundaries 1–4, 9–10, 13, 16	
rad.fepdiff	rad.fepdiffd	1	Fractional emissive power	Boundaries 1–4, 9–10, 13, 16	
rad.T_band	rad.Td_band	K	Temperature	Boundaries 1–4, 9–10, 13, 16	
rad.T	rad.T_band	K	Temperature	Boundaries 1–4, 9–10, 13, 16	
rad.Trad	rad.dsurf2.minput_ temperature	K	Temperature	Boundaries 1–4, 9–10, 13, 16	
rad.Gm_gp	gpeval(2,rad.Gm)	W/m²	Mutual surface irradiation	Boundaries 1–4, 9–10, 13, 16	
rad.Gmu_gp	0	W/m²	Mutual surface irradiation, upside	Boundaries 1–4, 9–10, 13, 16	
rad.Gmd_gp	gpeval(2,rad.Gmd)	W/m²	Mutual surface irradiation, downside	Boundaries 1–4, 9–10, 13, 16	
rad.Famb_gp	gpeval(2,rad.Famb)	1	Ambient view factor, Gauss point evaluation	Boundaries 1–4, 9–10, 13, 16	
rad.Fambu_gp	0	1	Ambient view factor, Gauss point evaluation, upside	Boundaries 1–4, 9–10, 13, 16	
rad.Fambd_gp	gpeval(2,rad.Famb d)	1	Ambient view factor, Gauss point evaluation, downside	Boundaries 1–4, 9–10, 13, 16	
rad.diru	0	1	Radiation direction, upside	Boundaries 1–4, 9–10, 13, 16	
rad.ebu	rad.nS2S^2*sigma_ const*rad.Tu_band ^4*rad.fepu	W/m²	Blackbody emissive power, upside	Boundaries 1–4, 9–10, 13, 16	

Name	Expression	Unit	Description	Selection	Details
rad.ebambu	rad.nS2S^2*sigma_const*rad.Tambu^4*rad.fepambu	W/m²	Blackbody emissive power, upside	Boundaries 1–4, 9–10, 13, 16	
rad.fepu	1+15*integrate(rad.zeta^3/(1-exp(rad.zeta)),rad.zeta,eps,eps,1.0E-6)/pi^4	1	Fractional emissive power, upside	Boundaries 1–4, 9–10, 13, 16	
rad.fepambu	1+15*integrate(rad.zeta^3/(1-exp(rad.zeta)),rad.zeta,eps,eps,1.0E-6)/pi^4	1	Fractional emissive power, upside	Boundaries 1–4, 9–10, 13, 16	
rad.fepdiffu	1+15*integrate(rad.zeta^3/(1-exp(rad.zeta)),rad.zeta,eps,eps,1.0E-6)/pi^4	1	Fractional emissive power, upside	Boundaries 1–4, 9–10, 13, 16	
rad.Tu_band	0.5*((1+rad.opaque)*rad.Tradu+(1-rad.opaque)*rad.Tradd)	K	Upside temperature	Boundaries 1–4, 9–10, 13, 16	
rad.Gambu	rad.Fambu*rad.epsilon_ambu_band*rad.ebambu	W/m²	Ambient irradiation, upside	Boundaries 1–4, 9–10, 13, 16	
rad.Gextu	rad.q0su	W/m²	External irradiation, upside	Boundaries 1–4, 9–10, 13, 16	+ operation
rad.Gradu	nojac(rad.Gmu+rad.Gambu+rad.Gextu)	W/m²	Surface irradiation, upside	Boundaries 1–4, 9–10, 13, 16	
rad.Ju	rad.Ju_band	W/m²	Surface radiosity, upside	Boundaries 1–4, 9–10, 13, 16	
rad.Ju_band	rad.dsurf2.Ju_band	W/m²	Surface radiosity, upside	Boundaries 1–4, 9–10, 13, 16	
rad.rfluxu	rad.rfluxu_band	W/m²	Radiative heat flux, upside	Boundaries 1–4, 9–10, 13, 16	
rad.rfluxu_band	0	W/m²	Radiative heat flux, upside	Boundaries 1–4, 9–10, 13, 16	

Name	Expression	Unit	Description	Selection	Details
rad.Idiff	0	W/m²	Diffuse irradiance	Boundaries 1–4, 9–10, 13, 16	
rad.Idiff_band	rad.Idiffd_band	W/m²	Diffuse irradiance	Boundaries 1–4, 9–10, 13, 16	
rad.Idiffu_band	0	W/m²	Diffuse irradiance, upside	Boundaries 1–4, 9–10, 13, 16	
rad.Idiffd_band	0	W/m²	Diffuse irradiance, downside	Boundaries 1–4, 9–10, 13, 16	
rad.Tambu	rad.Tamb	K	Ambient temperature, upside	Boundaries 1–4, 9–10, 13, 16	
rad.dird	-1	1	Radiation direction, downside	Boundaries 1–4, 9–10, 13, 16	
rad.ebd	rad.nS2S^2*sigma_const*rad.Td_band^4*rad.fepd	W/m²	Blackbody emissive power, downside	Boundaries 1–4, 9–10, 13, 16	
rad.ebambd	rad.nS2S^2*sigma_const*rad.Tambd^4*rad.fepambd	W/m²	Blackbody emissive power, downside	Boundaries 1–4, 9–10, 13, 16	
rad.fepd	1+15*integrate(rad.zeta^3/(1-exp(rad.zeta)),rad.zeta,eps,eps,1.0E-6)/pi^4	1	Fractional emissive power, downside	Boundaries 1–4, 9–10, 13, 16	
rad.fepambd	1+15*integrate(rad.zeta^3/(1-exp(rad.zeta)),rad.zeta,eps,eps,1.0E-6)/pi^4	1	Fractional emissive power, downside	Boundaries 1–4, 9–10, 13, 16	
rad.fepdiffd	1+15*integrate(rad.zeta^3/(1-exp(rad.zeta)),rad.zeta,eps,eps,1.0E-6)/pi^4	1	Fractional emissive power, downside	Boundaries 1–4, 9–10, 13, 16	
rad.Td_band	0.5*((1+rad.opaque)*rad.Tradd+(1-rad.opaque)*rad.Tradu)	K	Downside temperature	Boundaries 1–4, 9–10, 13, 16	

Name	Expression	Unit	Description	Selection	Details
rad.Gambd	rad.Fambd*rad.epsilon_ambd_band*rad.ebambd	W/m²	Ambient irradiation, downside	Boundaries 1–4, 9–10, 13, 16	
rad.Gextd	rad.q0sd	W/m²	External irradiation, downside	Boundaries 1–4, 9–10, 13, 16	+ operation
rad.Gradd	nojac(rad.Gmd+rad.Gambd+rad.Gextd)	W/m²	Surface irradiation, downside	Boundaries 1–4, 9–10, 13, 16	
rad.Jd	rad.Jd_band	W/m²	Surface radiosity, downside	Boundaries 1–4, 9–10, 13, 16	
rad.Jd_band	rad.dsurf2.Jd_band	W/m²	Surface radiosity, downside	Boundaries 1–4, 9–10, 13, 16	
rad.rfluxd	rad.rfluxd_band	W/m²	Radiative heat flux, downside	Boundaries 1–4, 9–10, 13, 16	
rad.rfluxd_band	rad.epsilond_band*(rad.Gradd-rad.ebd)	W/m²	Radiative heat flux, downside	Boundaries 1–4, 9–10, 13, 16	
rad.Tambd	rad.Tamb	K	Ambient temperature, downside	Boundaries 1–4, 9–10, 13, 16	
rad.epsilon	rad.epsilon_band	1	Emissivity	Boundaries 1–4, 9–10, 13, 16	
rad.epsilonu	subst(material.epsilon_rad,rad.dsurf2.minput_temperature,rad.Tradu,rad.dsurf2.minput_length,rad.lambda)	1	Emissivity, upside	Boundaries 1–4, 9–10, 13, 16	Meta
rad.epsilond	subst(material.epsilon_rad,rad.dsurf2.minput_temperature,rad.Tradd,rad.dsurf2.minput_length,rad.lambda)	1	Emissivity, downside	Boundaries 1–4, 9–10, 13, 16	Meta
rad.epsilon_band	0.5*((1+rad.opaque)*rad.epsilond_band+(1-rad.opaque)*rad.ep	1	Emissivity	Boundaries 1–4, 9–10, 13, 16	

Name	Expression	Unit	Description	Selection	Details
	silonu_band)				
rad.epsilonu_band	integrate(rad.epsilonu,rad.lambda,0[m]*(1+0.25*eps),rad.lambdainf*(1-0.25*eps),1.0E-6)/max(rad.lambdainf*(1-0.25*eps)-0[m]*(1+0.25*eps),eps)	1	Emissivity, upside	Boundaries 1–4, 9–10, 13, 16	
rad.epsilond_band	integrate(rad.epsilond,rad.lambda,0[m]*(1+0.25*eps),rad.lambdainf*(1-0.25*eps),1.0E-6)/max(rad.lambdainf*(1-0.25*eps)-0[m]*(1+0.25*eps),eps)	1	Emissivity, downside	Boundaries 1–4, 9–10, 13, 16	
rad.epsilon_amb	rad.epsilon_amb_band	1	Ambient emissivity	Boundaries 1–4, 9–10, 13, 16	
rad.epsilon_ambu	1	1	Ambient emissivity, upside	Boundaries 1–4, 9–10, 13, 16	
rad.epsilon_ambd	1	1	Ambient emissivity, downside	Boundaries 1–4, 9–10, 13, 16	
rad.epsilon_amb_band	0.5*((1+rad.opaque)*rad.epsilon_ambd_band+(1-rad.opaque)*rad.epsilon_ambu_band)	1	Ambient emissivity	Boundaries 1–4, 9–10, 13, 16	
rad.epsilon_ambu_band	integrate(rad.epsilon_ambu,rad.lambda,0[m]*(1+0.25*eps),rad.lambdainf*(1-0.25*eps),1.0E-6)/max(rad.lambdainf*(1-0.25*eps)-0[m]*(1+0.25*eps),eps)	1	Ambient emissivity, upside	Boundaries 1–4, 9–10, 13, 16	
rad.epsilon_ambd_band	integrate(rad.epsilon_ambd,rad.lambda,0[m]*(1+0.25*e	1	Ambient emissivity, downside	Boundaries 1–4, 9–10, 13, 16	

Name	Expression	Unit	Description	Selection	Details
	ps),rad.lambdainf*(1-0.25*eps),1.0E-6)/max(rad.lambdainf*(1-0.25*eps)-0[m]*(1+0.25*eps),eps)				

Shape functions

Name	Shape function	Unit	Description	Shape frame	Selection	Details
rad.dsurf2.Ju_band	Lagrange (Linear)	W/m²	Surface radiosity, upside	Spatial	Boundaries 1–4, 9–10, 13, 16	
rad.dsurf2.Ju_band	Lagrange (Linear)	W/m²	Surface radiosity, upside	Spatial	Edges 1–4, 9–11, 15–16, 21–22, 24	Slit
rad.dsurf2.Jd_band	Lagrange (Linear)	W/m²	Surface radiosity, downside	Spatial	Boundaries 1–4, 9–10, 13, 16	
rad.dsurf2.Jd_band	Lagrange (Linear)	W/m²	Surface radiosity, downside	Spatial	Edges 1–4, 9–11, 15–16, 21–22, 24	Slit

Weak Expressions

Weak expression	Integration order	Integration frame	Selection
((1-rad.epsilond_band)*rad.Gradd+rad.epsilond_band*rad.ebd-rad.Jd_band)*test(rad.Jd_band)	2	Spatial	Boundaries 1–4, 9–10, 13, 16

Constraints

Constraint	Constraint force	Shape function	Selection	Details
rad.Ju_band-rad.Jd_band	test(rad.dsurf2.Ju_band)	Lagrange (Linear)	Boundaries 1–4, 9–10, 13, 16	Elemental

2.5 GRID 1

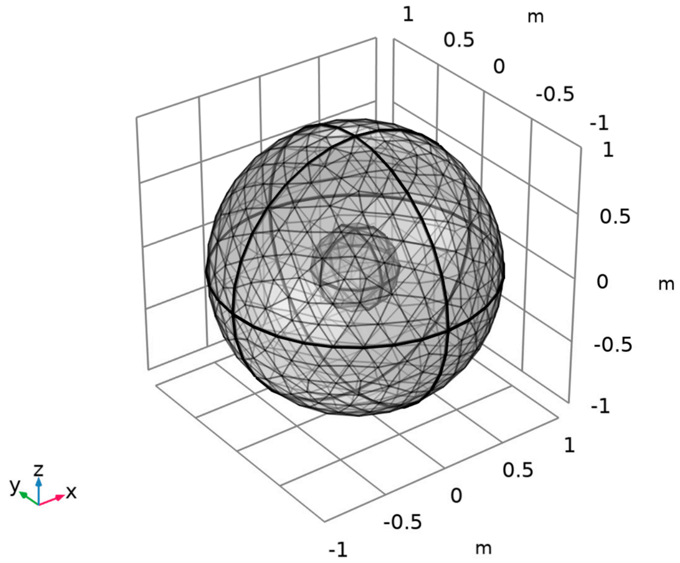

grid 1

MESH STATISTICS

Description	Value
Status	Complete mesh
Mesh vertices	486
Triangles	964
Edge elements	132
Vertex elements	12
Number of elements	964
Minimum element quality	0.5757
Average element quality	0.8577
Element area ratio	0.16837
Mesh face area	13.55 m^2

2.5.1 size (size)

SETTINGS

Description	Value
Maximum element size	0.2
Minimum element size	0.036
Curvature factor	0.6
Resolution of narrow regions	0.5
Maximum element growth rate	1.5

2.5.2 free triangular meshes 1 (ftri1)

SELECTION

Geometric entity level	Boundary
Selection	Geometry geom1: Dimension 2: Boundaries 1–16

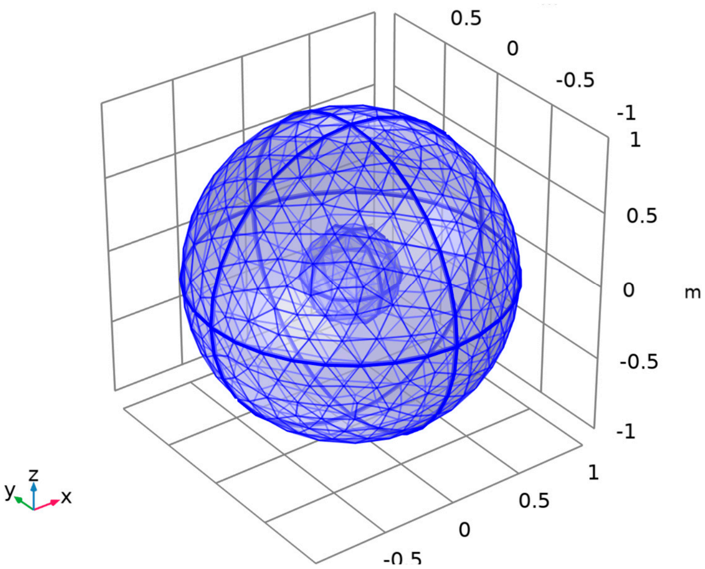

free triangular meshes 1

SETTINGS

Description	Value
Last build time	1
Built with	COMSOL 6.1.0.252 (win64)|2023 - 09 - 13T12:18:03.408516500

3 study 1

COMPUTATION INFORMATION

Computation time	

3.1 STEADY STATE

STUDY SETTINGS

Description	Value
Include geometric nonlinearity	Off

PHYSICS AND VARIABLES SELECTION

Physics interface	Discretization
Surface-to-surface radiation (rad)	physics

MESH SELECTION

Geometry	Mesh
Geometry 1 (geom1)	mesh1

3.2 SOLVER CONFIGURATIONS

3.2.1 solution 1

Compile Equations: Steady state (st1)

STUDY AND STEP

Description	Value
Use study	Study1
Use study step	Steady state

LOG

Dependent variable 1 (v1)

GENERAL

Description	Value
Defined by study step	Steady state

LOG

Surface Radiance, lower side (comp1.rad.dsurf1.Jd_band) (comp1_rad_dsurf1_Jd_band)

GENERAL

Description	Value
Field components	comp1.rad.dsurf1.Jd_band

SCALING

Description	Value
Method	Initial value based

Surface Radiance, upper side (comp1.rad.dsurf1.Ju_band) (comp1_rad_dsurf1_Ju_band)

GENERAL

Description	Value
Field components	comp1.rad.dsurf1.Ju_band

SCALING

Description	Value
Method	Initial value based

Surface Radiance, lower side (comp1.rad.dsurf2.Jd_band) (comp1_rad_dsurf2_Jd_band)

GENERAL

Description	Value
Field components	comp1.rad.dsurf2.Jd_band

SCALING

Description	Value
Method	Initial value based

Surface Radiance, upper side (comp1.rad.dsurf2.Ju_band) (comp1_rad_dsurf2_Ju_band)

GENERAL

Description	Value
Field components	comp1.rad.dsurf2.Ju_band

SCALING

Description	Value
Method	Initial value based

Steady state solver 1 (s1)

GENERAL

Description	Value
Defined by study step	Steady state

Separation 1 (se1)

GENERAL

Description	Value
Tolerance factor	0.1

Description	Value
Stabilization and acceleration	Anderson acceleration

Radiation (ss1)

GENERAL

Description	Value
Variables	{Surface Radiance, upper side (comp1.rad.dsurf1.Ju_band), Surface Radiance, lower side (comp1.rad.dsurf1.Jd_band), Surface Radiance, upper side (comp1.rad.dsurf2.Ju_band), Surface Radiance, lower side (comp1.rad.dsurf2.Jd_band)}
Linear solver	Direct radiation variable

METHOD AND TERMINATION

Description	Value
Damping factor	0.8

Direct radiation variable (d1)

GENERAL

Description	Value
Solver	PARDISO
Pivoting perturbation	1E-13

4 results

4.1 DATA SET

4.1.1 study 1/solution 1

SOLUTION

Description	Value
Solution	Solution 1
Component	Component 1 (comp1)

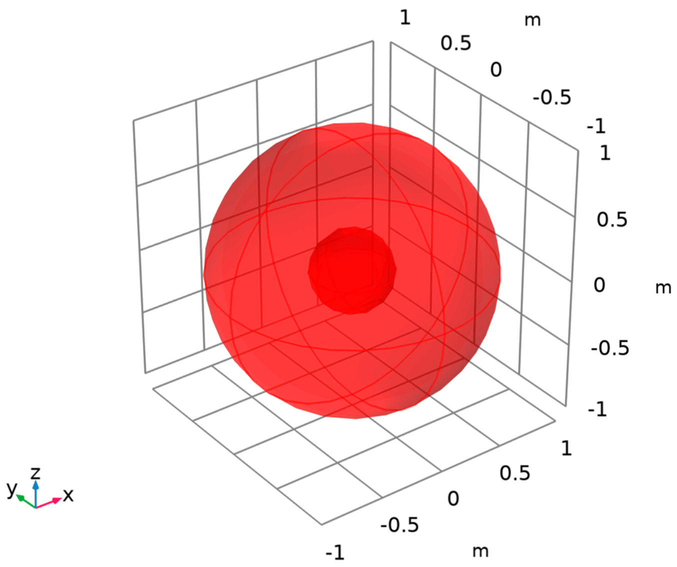

Dataset: Study 1/Solution 1

4.2 DERIVED VALUES

4.2.1 global calculation 1

OUTPUT

Evaluated in	Form 1

DATA

Description	Value
Dataset	Study 1/Solution 1

EXPRESSIONS

Expression	Unit	Description
intop_int(comp1.rad.radopu(int,0))/intop_int(1)		Internal-to-internal perspective coefficient

Expression	Unit	Description
intop_ext(comp1.rad.radopd(int,0))/intop_int(1)		Internal to external perspective coefficient
intop_ext(comp1.rad.radopd(0,ext))/intop_ext(1)		External to external perspective coefficient
intop_int(comp1.rad.radopu(0,ext))/intop_ext(1)		Exterior-to-interior perspective coefficient

4.3 Form

4.3.1 Form 1

Global computation 1

Time	Internal-to-internal perspective coefficient	Internal to external perspective coefficient	External to external perspective coefficient	Exterior-to-interior perspective coefficient
0	0	0.99948	0.91379	0.086213
